# iBAG: integrative Bayesian analysis of high-dimensional multiplatform genomics data

**DOI:** 10.1093/bioinformatics/bts655

**Published:** 2012-11-09

**Authors:** Wenting Wang, Veerabhadran Baladandayuthapani, Jeffrey S. Morris, Bradley M. Broom, Ganiraju Manyam, Kim-Anh Do

**Affiliations:** ^1^Department of Biostatistics and ^2^Department of Bioinformatics and Computational Biology, The University of Texas, MD Anderson Cancer Center, Houston, TX 77030, USA

## Abstract

**Motivation:** Analyzing data from multi-platform genomics experiments combined with patients’ clinical outcomes helps us understand the complex biological processes that characterize a disease, as well as how these processes relate to the development of the disease. Current data integration approaches are limited in that they do not consider the fundamental biological relationships that exist among the data obtained from different platforms.

**Statistical Model:** We propose an integrative Bayesian analysis of genomics data (iBAG) framework for identifying important genes/biomarkers that are associated with clinical outcome. This framework uses hierarchical modeling to combine the data obtained from multiple platforms into one model.

**Results:** We assess the performance of our methods using several synthetic and real examples. Simulations show our integrative methods to have higher power to detect disease-related genes than non-integrative methods. Using the Cancer Genome Atlas glioblastoma dataset, we apply the iBAG model to integrate gene expression and methylation data to study their associations with patient survival. Our proposed method discovers multiple methylation-regulated genes that are related to patient survival, most of which have important biological functions in other diseases but have not been previously studied in glioblastoma.

**Availability:** http://odin.mdacc.tmc.edu/∼vbaladan/.

**Contact:** veera@mdanderson.org

**Supplementary information:** Supplementary data are available at *Bioinformatics* online.

## 1 INTRODUCTION

The overarching goal of cancer genomics is to customize patient care decisions according to diverse genetic and epigenetic alterations for a tumor (Chin *et al.*, 2011; Vogelstein and Kinzler, 1993; Weir *et al.*, 2004). Early cancer genomics studies focused on only a single type of alteration at a time to assess these changes, e.g. high-resolution copy number profiling led to the discovery of novel oncogenes in ovarian cancer (Nanjundan *et al.*, 2007), melanoma (Scott *et al.*, 2009) and lung carcinoma (Bass *et al.*, 2009). Some of these findings have already been translated into personalized cancer treatment, such as imatinib for KIT-mutated gastrointestinal stromal tumors (Handolias *et al.*, 2010) and trastuzumab for HER2-positive breast tumors (Pegram and Slamon, 2000).

As technologies to perform comprehensive profiling of the cancer genome have progressed, different technology platforms, from basic capillary electrophoresis sequencing to advanced forms of microarrays, have been brought together on the same patient set. For example, the Cancer Genome Atlas (TCGA) is a worldwide research program that currently encompasses comprehensive genomic datasets for >20 types of cancer (http://cancergemone.nih.gov; Hudson *et al.*, 2010). The work of TCGA is motivating approaches for integrating data outputs from different types of technology platforms to identify important biomarkers related to cancer development and progression. The key hypothesis behind these approaches is that cancer consists of hundreds of distinct molecular changes, from multiple types of genetic and epigenetic alterations to the interactions among them. Each type of alteration provides a different and complementary view of the whole genome. Hence, integrating multiple aspects of the genome and the underlying biological processes to identify novel targets is essential and has the potential to improve the clinical management of cancer.

The concept of integration is very broad (see review by Hamid *et al.*, 2009). Such integration studies can be divided into three general groups according to the primary focus of the study (Daemen *et al.*, 2009). The focus of the first group, called *sequential integration* studies, is the sequential analysis of heterogeneous data from different platforms for the purpose of understanding the biological evolution of disease as opposed to predicting clinical outcome (Fridlyand *et al.*, 2006; Qin, 2008; Tomioka *et al.*, 2008). In this group, data obtained on one type of platform are analyzed along with the clinical outcome data, and then a second data platform is subsequently used to clarify or confirm the results obtained from the first platform. For example, Qin (2008) showed that microRNA expression can be used to sort tumors from normal tissues, regardless of tumor type. The study then analyzed the relationship between the candidate target genes for the cancer-related microRNAs and mRNA expression and disease status.

The focus of the second group of integration studies, which we call *biological integration* studies, is the analysis of biological pathways and regulatory mechanisms among data obtained from different platforms, such as the relationship between gene expression and protein abundances, or the relationship between gene expression and copy number changes in patient tumor samples (Karpenko and Dai, 2010; van Wieringen *et al.*, 2012; Waters *et al.*, 2006). The challenge for this group of studies is that the biological annotation databases used for mapping different datasets are inconsistent. An R-package to match array comparative genomic hybridization (CGH) and gene expression microarray features for integrative analysis purposes was provided by van Wieringen *et al.* (2012).

The focus of the third group of integration studies, which we term *model-based integration* studies, is the analysis of data obtained from multiple platforms that are combined into one statistical model to identify clinically relevant genes and/or to predict clinical outcome. Instead of merging datasets or analyzing them sequentially, the data from different platforms are treated equally, and the most relevant features are selected from all available platforms (Daemen *et al.*, 2009; Lanckriet *et al.*, 2004). For example, Daemen *et al.* (2009) proposed a kernel-based approach to integrate data from multiple platforms for the classification of discrete clinical outcomes. They showed that the area under curve (AUC) based on integrated data used for predictions was significantly improved compared with the AUC based on data from a single platform. However, these studies treated each platform independently and ignored the underlying biological mechanisms among different platforms. Witten and Tibshirani (2009) developed a supervised canonical correlation model to find significant axes of correlations between multiple multivariate datasets at a global (chromosomal) level. They integrated copy number and gene expression data and identified linear combinations (canonical variables) that are related to a clinical outcome. However, they also did not take the biological mechanisms (directionality) into account, as we detail later in the text.

Our proposed method takes a different approach in modeling biological relationships among molecular features measured by different platforms, by focusing on relationships at a ‘gene-centric’ level. We first study the underlying biological mechanisms, relating the data across the different platforms. Then using this information, we partition gene expression into different (independent) units and use this to identify genes relevant to clinical outcome as modulated by these different platforms. We hypothesize (and show) that, compared with non-integrative methods, our proposed method can detect clinically relevant gene expression changes with greater power and a lower false discovery rate (FDR), in addition to obtaining results that are more biologically interpretable.

**Fig. 1.**
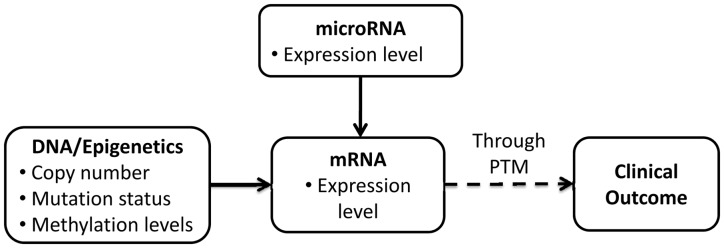
Associations among different molecular features and with clinical outcome. PTM: post-translational modification; solid (dashed) arrow: products from one platform are influenced directly (indirectly) by the products from the other platform

Molecular biology has shown that features identified on different platforms influence clinical outcome at different levels. For example, in TCGA studies, copy number, methylation, mutation status, mRNA expression, microRNA expression and the expression of proteins in specific signaling pathways have been measured on the same set of samples. The fundamental biological relationships among the products of these different platforms and their associations with clinical outcome are shown in Figure 1. Generally speaking, molecular features measured at the transcript level (e.g. mRNA expression) affect clinical outcome more directly than molecular features measured at the DNA/epigenetics level (e.g. copy number, methylation and mutation status). Molecular features measured at the DNA level affect clinical outcome by influencing mRNA expression (Fabiani *et al.*, 2010; Glinsky, 2006; de Tayrac *et al.*, 2009). Similarly, microRNAs, post-transcriptional regulators that bind to complementary sequences on target mRNAs, influence mRNA through translational repression or target degradation, which then affects clinical outcome (Tseng *et al.*, 2011).

Conducting the proposed integrative analysis is a challenge because of the complicated biological relationships and the different intrinsic structures of various platforms. For example, molecular features measured at the DNA level regulate the mRNA expressions of the corresponding genes or nearby genes (Peng *et al.*, 2010). In contrast, microRNAs can regulate the mRNA expression of any gene, regardless of its locus, and each microRNA molecule has multiple target genes. Another challenge underlying this analysis is the large scale of the different types of gene alterations in contrast to the limited number of patient samples for such a study. Hence, an easily implemented and efficient variable selection method is needed for such an integration analysis.

We have developed the integrative Bayesian analysis of genomics data (iBAG) model to address these challenges. The main advantages of our proposed model can be summarized as follows. The iBAG model (i) uses a hierarchical approach to model the fundamental biological relationships underlying molecular features obtained by different platforms; (ii) accounts for both the influences of different platforms and the biological relationships among the platforms in one unified model to predict patients’ clinical outcomes; (iii) can conduct high-dimensional variable selection, which adapts to analyzing hundreds of distinct molecular entity effects jointly in one model; (iv) uses a Bayesian framework, which allows the model enough flexibility to estimate the different intrinsic structures of biological relationships for different high-throughput platforms; and (v) is computationally efficient and feasible owing to its closed forms of full conditional posterior distributions for posterior sampling.

The rest of this article is organized as follows. In Section 2, we describe the iBAG model construction along with prior formulations for continuous, discrete and survival clinical outcomes. In Section 3, we introduce an innovative approach for conducting high-dimensional variable selection using Bayesian FDRs. In Section 4, we illustrate the performance of the iBAG model and use simulations to compare its performance with those of alternative approaches. In Section 5, we apply the iBAG model to integrate gene expression and methylation data for TCGA’s glioblastoma study, and evaluate the associations between those data and patients’ survival times. Finally, we provide a summary and discussion in Section 6. The technical details and additional simulation results are contained in the Supplementary Material (Section S1).

## 2 THE iBAG MODEL

For ease of interpretation and exposition, we illustrate our methodology using two platforms at a time—DNA methylation and gene expression data. Integration across more than two platforms can be done in an analogous manner, as discussed in Section 6.

### 2.1 Model for continuous outcome

Suppose the total number of patients is *N*. For the *n*th patient, our observed datum consists of—(i) *Y_n_*, the clinical outcome of interest [e.g. survival time, tumor(sub)type], (ii) 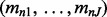, the measures of methylation levels for *J* probes/sites on the whole genome, (iii) 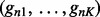, the measures of gene expression level for *K* genes, and (iv) 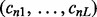, the values of *L* clinical (non-genomic) factors (e.g. tumor stage, age and other demographic variables). Hence, all of the observed datasets in our study can be denoted (in matrix notation) as 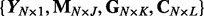.

We propose the following two-component hierarchical construction for our iBAG model: a *mechanistic* model to infer direct effects of methylation on gene expression, and a *clinical* model that uses this information to predict a clinical outcome. The first component of our model assesses the underlying biological relationship between methylation and gene expression. The expression level of a gene is affected primarily by the methylation sites in the promoter region and is usually lower when its promoter is highly methylated. However, methylation is only one of the many potential factors contributing to a change in gene expression level (as shown in Fig. 1). The mechanistic model regresses the measure of gene expression for the *k*th gene (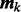) on the methylation measures obtained within the promoter of the *k*th gene. To match the methylation sites to a given gene, we use the annotation files for the platforms and use those methylation sites that are encompassed within the promoter region of a given gene—thus potentially allowing multiple methylation sites to map to a particular gene. The second component of our model assesses when the expression of a particular gene affects the clinical outcome, whether this effect is modulated through methylation and/or through some other mechanisms that are independent of methylation (e.g. microRNA, copy number effects). (1)
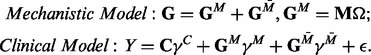



The parameters of the mechanistic and clinical models have the following interpretations:

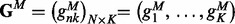
, where 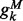 denotes the part of the expression changes of the *k*th gene expression feature that is modulated through methylation (**M**).
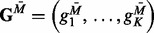
, where 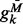 is an *N* × 1 vector that denotes the part of the expression changes of the *k*th gene expression feature that is modulated by mechanisms other than methylation (e.g. microRNA, copy number effects). We assume that 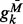 follows a multivariate normal distribution with mean 0 and covariance matrix 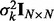 for 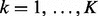.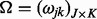, where ω*_jk_* is the ‘gene-methylation’ effect that estimates the (conditional) effect of the *j*th methylation feature on the *k*th feature identified from the gene expression data.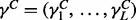, where 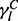 denotes the effect of the *l*th clinical factor on clinical outcome ***Y***.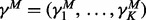, where 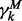 estimates the effect of 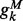 on ***Y***, which can be interpreted as the effect of gene expression modulated by methylation for the *k*th feature identified from the gene expression data. We denote this partial effect of gene expression on clinical outcome as a *type M effect*.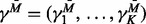, where 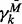 measures the effect of 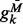 on ***Y***, which can be interpreted as the effect of gene expression modulated by other sources for the *k*th feature identified from the gene expression data. We denote this partial gene expression effect on clinical outcome as a *type *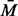 *effect*.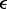 is the error term that accounts for variation not explained by the observed genomic and clinical factors and which is assumed to follow a normal distribution with a common standard deviation σ.

**Fig. 2.**
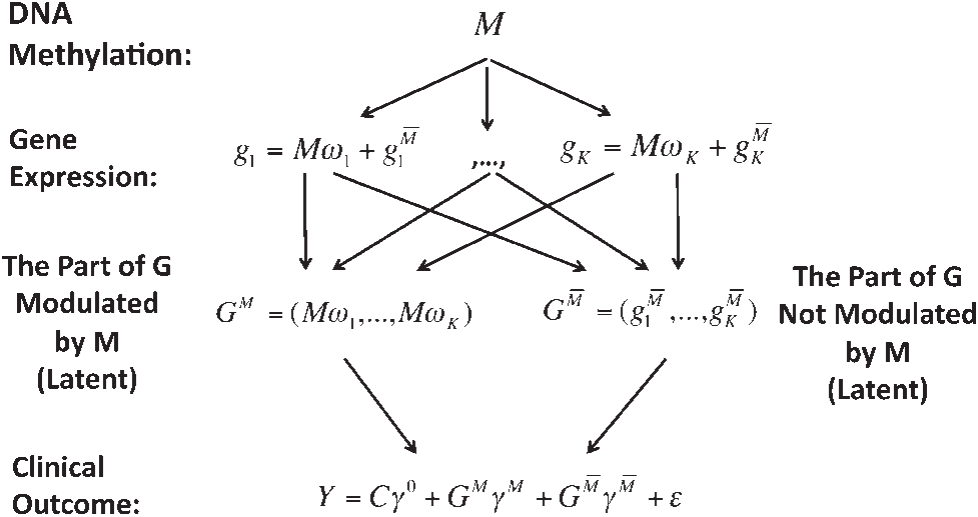
Graphical representation of the structure of the iBAG model

In essence, our mechanistic model divides the gene expression levels into two components—one modulated by methylation 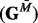 and the other independent of methylation 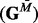
—and uses both of these components (jointly) in the clinical model for the prediction of relevant outcomes. Figure 2 further exemplifies the architecture of the iBAG model for integrating data from two platforms. The formal directed acyclic graphical representation is given in Supplementary Fig. S1.

### 2.2 Prior construction

There are various univariate and multivariate approaches for fitting the iBAG model, as specified above, which require some variable selection and/or sparsity to regularize the ill-posed high-dimensional problem—as both the number of genes (*K*) and methylation features (*J*) are potentially of very high dimension (on the level of thousands) as compared with the sample size (*N* = hundreds), and most of the genes are expected to have very weak effects on clinical outcome. We use a Bayesian penalized regression approach that not only jointly models the mechanistic and clinical components in Equation (1) but also provides a natural approach for imposing sparsity and performing variable selection via hierarchical priors.

We denote our full parameter set as 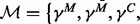 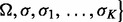 and specify our prior construction for each of these parameters. To model the main constructs of interest, 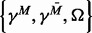, we use the Bayesian formulation of the lasso (Tibshirani, 1996), which serves a dual purpose. First, similar to the lasso regression with *L*_1_ penalty, it achieves sparsity (variable selection) via non-linear shrinkage of small/weak effects toward zero. This approach has proven to be useful in identifying genomic features with large effects on clinical outcomes in various genomic studies (Hoggart *et al.*, 2008; Li *et al.*, 2011). Second, and more importantly, as the complete conditionals are available in closed forms, the Bayesian formation of the lasso substantially aids our Bayesian computations for large genomic datasets such as those considered here. Specifically, we can write the double exponential (lasso) prior distribution as a scale mixture of a normal distribution with an exponential mixing density (Park and Casella, 2008), which allows us to use Gibbs sampling to draw the samples from the posterior distribution.

Thus our (conditional) Bayesian lasso prior for the type *M* effects (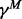) can be written as 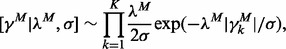

where λ*^M^* is the shrinkage parameter for the vector 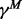, and σ is the standard deviation for the random error term 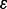. Similarly, we define a Bayesian lasso prior for 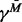, conditioned on the (different) shrinkage parameter 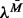 and the (same) standard deviation σ.

For 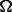, which models the gene-methylation effects in the mechanistic model, we adopt the following strategy. When the number of features matching a given gene (promoter) is lower than the sample size (*N*) (e.g. methylation features), we assume that ω*_jk_* follows a normal distribution if 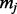 is within the *k*th gene promoter and ω*_jk_* = 0, otherwise. In cases where the number of probes/features exceeds *N* for a particular gene (e.g. microRNA features), we allow for a Bayesian lasso prior, as described earlier in the text, to achieve regularization.

For 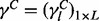, which models the effects of clinical factors, we simply assume that the prior of each 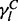 is a multivariate normal distribution with mean 0 and large variance (e.g. on the order of 10^6^ for a variable with standard deviation = 1). For the error variance (σ^2^ and 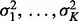), we assume an improper prior 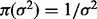. For other hyper parameters in the hierarchical model, we assume a gamma prior on 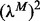 and 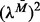, with mean parameter α*^M^*, 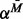 and scale parameter ξ*^M^*, 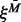, respectively. In our applications, we assume the values of α*^M^*, 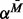, ξ*^M^* and 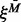 are all equal to 1.

### 2.3 Estimation via Markov chain Monte Carlo

Our complete iBAG model can be expressed hierarchically as 
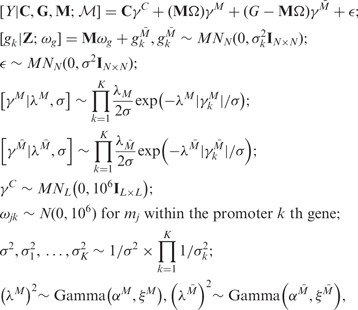

where 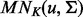 denotes the *K* dimensional multivariate normal distribution with mean ***u*** and covariance matrix Σ.

To conduct estimation and subsequent inference, we follow a fully Bayesian analysis of the iBAG model specified above using Markov chain Monte Carlo (MCMC) approaches (Casella and George, 1992). Specifically, we iteratively draw posterior samples from the full conditional distributions of the parameter sets, as specified below.

#### 2.3.1 Mechanistic model parameters



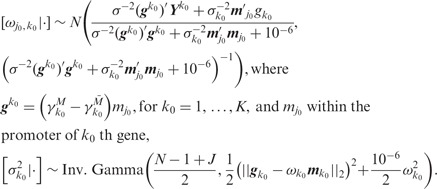



#### 2.3.2 Clinical model parameters



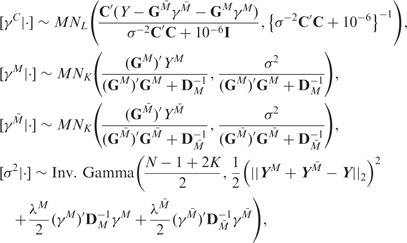



where 
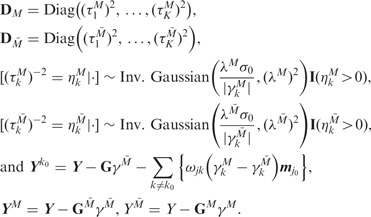



#### 2.3.3 Shrinkage parameters



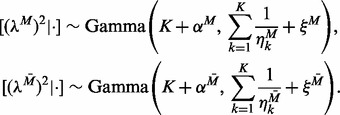



As all the above full conditional likelihoods are available in closed form, an efficient Gibbs sampler can be used to update our posterior distributions by drawing samples sequentially from full conditional distributions for each parameter set. See details in Supplementary Material (Section S1).

### 2.4 iBAG model for discrete and censored outcomes

The construction of the iBAG model can be easily extended to model discrete and censored outcomes using latent variable formulations (Albert and Chib, 1993). Specifically, when ***Y*** is a binary variable taking values of 0 or 1 [e.g. tumor-(sub)type], we use a probit latent-variable formulation that preserves all the conjugate constructions. We let ***Z*** be the (unobserved) latent variable that relates to ***Y*** as follows, 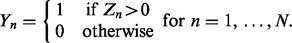



Then conditionally (on ***Z***) our iBAG model for discrete responses is the same as that shown in Equation (1), with ***Y*** in the clinical model replaced by ***Z*** and parameter representations and corresponding interpretations remaining exactly the same as those for continuous outcomes.

If the clinical outcome of interest is patient survival time (with censoring), we use the accelerated failure time model with a data augmentation approach (Tanner and Wong, 1987) to impute the censored values for this study. We let 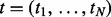 denote the survival time and 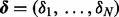 denote the censoring status. Still, we let 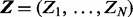 denote an unobserved latent variable. Given the latent variable ***Z*** for right-censored responses, the expression of the iBAG model is similar to Equation (1), changing the response variable ***Y*** to ***Z***.

The relationship between clinical outcome (*t_n_*,δ*_n_*) and the latent variable *Z_n_* can be expressed as 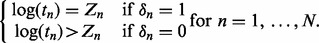



The full conditionals and the MCMC sampling schemes for discrete and survival responses are provided in the Supplementary Material (Section S2).

## 3 GENE SELECTION VIA FDRs

Our posterior sampling schemes for the iBAG model result in MCMC samples for all model parameters and, of specific interest to our study, the effects of gene expression levels on clinical outcomes modulated by and independent of methylation 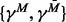. One key issue is to summarize this information in the MCMC samples to conduct gene selection. Typical inferential approaches, such as selection based on posterior quantiles or the maximum a posteriori (MAP) via MCMC samples, suffer from two drawbacks. First, the Bayesian lasso has excellent shrinkage properties but does not conduct natural model/variable selection, as it does not set the effects exactly equal to 0 (owing to an absolutely continuous prior). Second, such inferential methods do not allow for the natural incorporation of FDR controls that are commonly used in high-dimensional settings (Benjamini and Hochberg, 1995; Storey and Tibshirani, 2003).

We propose an alternative approach to obtaining posterior probabilities to evaluate the significance of gene expression effects that facilitates efficient FDR-based inferences. Let 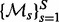 denote the *S* MCMC posterior samples for the model parameters. When the clinical outcome ***Y*** is continuous, for each MCMC sample, we compute the (conditional) MAP estimate of 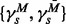, conditional on all other model parameters that can be obtained by minimizing the following objective/loss function: (2)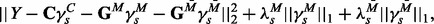 where 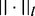 is the *l*-norm, and ***Y*** is the observed continuous outcome. When the clinical outcome is discrete or censored, the MAP estimate of 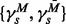 can be obtained similarly by replacing ***Y*** in Equation (2) with the MCMC samples for the unobserved latent variable ***Z***. Equation (2) is similar to the penalized objective function in the frequentist *lasso* (Tibshirani, 1996) with two different shrinkage parameters (λ*^M^*, 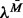)—however, with the key difference that it conditions on all the other model parameters, thus accounting for uncertainty. There are several algorithms available for computing the MAP estimate. We use the computationally efficient least angle regression selection algorithm (Efron *et al.*, 2004) to compute the estimates. We denote the resulting (conditional) estimates as 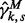 and 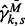 for the *k*th gene feature. Finally, we estimate the posterior probability of significance (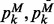) by computing the (empirical) frequencies of the non-zero elements in the MAP estimates for each gene *k* as

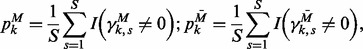
 where 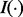 is the indicator function.

Note that, in this framework, 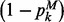 and 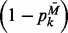 can be interpreted as estimates of the ‘local’ FDR or Bayesian *q*-values (Newton *et al.*, 2004; Storey and Tibshirani, 2003). Thus, given a desired global FDR α, we can determine a threshold ϕ_α_ to use in flagging the set of genes 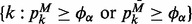 as significant genes associated with the clinical outcome. The significant threshold ϕ_α_ can be determined according to the method proposed by Morris *et al.* (2008). Let 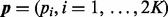 be the combined vector of posterior probabilities for 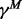 and 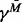. We then sort *p_i_* in descending order to obtain *p*_(_*_i_*_)_. Then 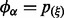, where 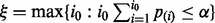. Using this cutoff, the expected proportion of genes found to be significant that are in fact false positive genes is α, in other words, we can control the average Bayesian FDR to be α.

## 4 SIMULATION STUDIES

In this section, we examine the operating characteristics of the proposed iBAG model through synthetic numerical examples. We use two versions of the iBAG model: an iBAG*_unified_* model as specified in Section 2 and an iBAG_2-_*_stage_* model in which the mechanistic and clinical models in Equation (1) are fit sequentially. Specifically, the mechanistic model involves fitting *K* linear regressions (for each gene separately). Subsequently, both the fitted values and the residuals from the mechanistic model are used as predictors in the clinical model. We use a similar lasso framework to estimate the clinical model and select genes related to clinical outcome. In addition, we compare the performance with those of two other models—a *non-integrative* (non-INT) model and a *single gene* (SG) model. In the non-INT model, we ignore the information provided by methylation and fit only the clinical model with gene expression features (**G**) as multivariate explanatory variables. In the SG model, we perform a multivariate linear regression for each gene separately with all of the genomic features available (including both mRNA and methylation levels) for the gene, to conduct selection based on individual *P*-values.

We simulate datasets that reflect the application dataset (analyzed in Section 5) as closely as possible. We fix the total number of patients at *N* = 200 and vary the total number of genes (*K* = 400, 600, 800, 1000). We assume that *J* = 200 out of *K* genes have had methylation levels measured (the proportion in the application dataset). Given the triplet, (*N, J, K*), we first generate methylation data, *m_nj_*, independently from Uniform (0,1), corresponding to the beta-values of DNA methylation used in the TCGA glioblastoma study (described in Section 5). Next, we simulate the gene expression values from a mixture of two normal distributions, based on the corresponding methylation measures, i.e. 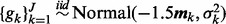 (regulated by methylation) and 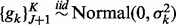 (not regulated by methylation). In the application dataset, ∼80% of the correlations between DNA methylations and the corresponding gene expression levels range from –0.4 to –0.8. To induce explicit dependence between methylation and gene expression, we assume 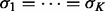 and vary the values (= 0.31, 0.44, 0.73) that respectively correspond to gene expression-methylation correlations 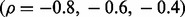. Finally, we use model (1) to generate the clinical outcomes ***Y*** by setting—(i) 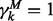 for 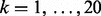 and 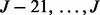, and 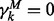 for all other *k*s; (ii) 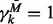 for 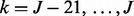 and 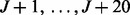, and 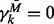 for all other *k*s and (iii) 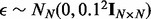. In essence, we have three groups of genes: group 1 consists of genes with only a nonzero type *M* effect, which is the gene expression effect modulated only by methylation (genes 1–20); group 2 consists of genes with only a non-zero type 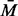 effect, which is the gene expression effect independent of methylation, but modulated by other mechanisms (gene *J* + 1 to gene *J* + 20); and group 3 consists of genes with both nonzero type *M* and type 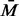 effects, i.e. the gene expression effects modulated (partially) by both methylation and other mechanisms (gene *J* − 21 to gene *J*). In total, we investigate 12 different data combinations based on variations of (*K*,ρ), and we generate 10 datasets for each combination.

**Fig. 3.**
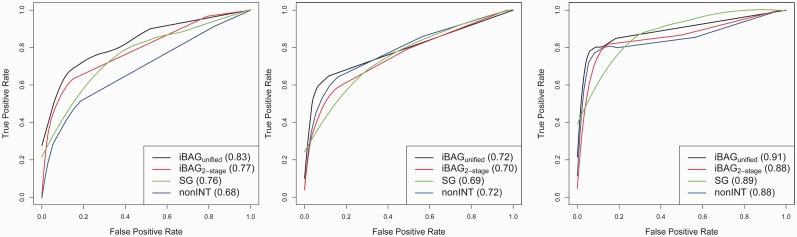
ROC curves of the true positive rate versus false positive rate of discovering genes in group 1 (genes with only non-zero type *M* effect; the left panel), group 2 (genes with only type 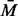 effect; the middle panel) and group 3 (genes with both type *M* and type 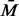 effects; the right panel) by the non-INT model, SG model, iBAG_2-_*_stage_* model and iBAG*_unified_* model when the total number of genes (*K*) is 1000, and the assumed correlation ρ between methylation and gene expression = –0.6 (values in parentheses are AUCs for the corresponding ROC curves)

For the non-INT and iBAG_2-_*_stage_* models, we use regular lasso regression and obtain receiver operating characteristic (ROC) curves by varying the shrinkage parameter. For the SG model, we vary the cutoff of the *P*-value for significance to obtain the ROC curves. For the iBAG*_unified_* model, we obtain the ROC curve by varying the significance threshold for the Bayesian posterior probabilities of the gene expression effects. We fit all four models, iBAG*_unified_*, iBAG_2-_*_stage_*, SG and non-INT, to all the simulated datasets and obtain ROC curves to identify the true effects of gene expression for the three groups of genes. For each model, we plot the means of the ROC curves based on the 10 simulated datasets for each (*K*,ρ) combination. For example, in Figure 3, we plot the ROC curves for identifying the true effects of gene expressions for the three groups of genes when *K* = 1000 and ρ = −0.6, which most closely mimics the real dataset analyzed in Section 5. Supplementary Table S1 shows the rank of performance for the four models in identifying the three groups of genes based on the areas under the ROC curves (AUC) values. Based on the AUC values, we can conclude that the iBAG*_unified_* model outperforms the non-INT, SG and iBAG_2-_*_stage_* models in identifying all three groups of genes. Although the iBAG_2-_*_stage_* model performs slightly worse than the non-INT model in identifying genes with only type 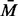 effects and genes with both type *M* and type 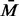 effects, it has a clear advantage in identifying genes with only type *M* effects. The SG model performs better than the non-INT model in identifying genes with only type *M* effects, but it has lower AUCs in identifying the other two groups of genes. The performances of the four models in the other 11 scenarios are similar (see Supplementary Figs S2.1–S2.3 for the detection of genes in groups 1–3).

Based on the results from all 12 scenarios, the performance of the four models can be summarized as follows: (i) Our proposed iBAG*_unified_* model consistently performs the best of all three models for discovering all three groups of genes; (ii) The iBAG_2-_*_stage_* model performs better than the non-INT model in discovering the genes in group 1, those with effects of gene expression modulated only by methylation; (iii) In discovering the genes in groups 2 and 3, the iBAG_2-_*_stage_* model performs as well as the non-INT model; and (iv) Compared with the non-INT model, the SG model performs better in identifying genes in group 1, but worse in identifying genes in the other two groups.

## 5 TCGA GLIOBLASTOMA MULTIFORME DATASET

Glioblastoma multiforme (GBM) is the most common and most aggressive type of malignant primary brain tumor in humans. The TCGA GBM dataset includes tumor samples from >500 patients with GBM, along with DNA copy number, mutation, methylation and gene expression information. Analyzing different platforms individually illuminates some of the pathobiologic features and molecular biomarkers in GBM. For example, Verhaak *et al.* (2010) proposed using gene expression data to develop clinically relevant molecular sub-classifications of GBM, and Noushmehr *et al.* (2010) used methylation levels to identify a subset of GBM tumors that harbor characteristic promoter DNA methylation alterations, referred to as the glioma CpG island methylator phenotype.

Here, we focus on integrating gene expression, methylation data and patients’ clinical features from the GBM study. The data can be downloaded directly from TCGA’s website (http://tcga-data.nci.nih.gov/tcga/tcgaHome2.jsp). The clinical outcome of interest is the overall survival time. The gene expression profile is obtained using Affymetrix Human Genome U133A Array. Level 2 data were downloaded from the TCGA website as of June 2011, and the data were normalized globally using BrainArray Custom Chip Definition Files (CDF) and the Robust Multichip Average (RMA) normalization method. Unsupervised hierarchical clustering (Pearson correlation and Ward linkage) and principal components analysis were used to search for batch effects, but no significant batch effects were observed. The DNA methylation information is obtained using the Illumina Human methylation 27 BeadChip. We directly downloaded the level 3 data from the TCGA website; there are no significant batch effects (http://bioinformatics.mdanderson.org/tcgabatcheffects/). For DNA methylation data, we use the beta value, which is a number between zero and one that measures the percentage of methylation. For the subsequent analyses, we briefly outline the data pre-processing steps here for the gene expression and methylation data. Complete details can be found in the Supplementary Material (Section S4.1). First, we filter out the under-expressed genes and the methylation features for which the beta values do not vary by patient. After this step, 7785 genes and 6890 methylation features remain. Second, we annotate the 6890 methylation features to the 7785 genes according to their positions on the chromosomes. Third, we choose the top genes based on univariate filtering, adjusting for patient age. Finally, 1000 genes (348 of them with methylation information available) remain for our analysis on 201 patients. Our goal is not only to understand methylation-based regulation of genes but also to use this information to find significant genes associated with survival times.

We randomly split the total data from 201 patients into a training dataset (data from 134 patients) and a test dataset (data from 67 patients). For the training dataset, we fit the following three models for the selected genes: (i) the non-INT model, with only gene expression information as explanatory variables, (ii) the additive (ADD) model, with both gene expression and methylation information as explanatory variables and assuming their effects on patients’ survival times are additive, (iii) the iBAG*_unified_* model for censored outcomes, which integrates both gene expression and methylation information. We include patient age as a clinical covariate for both the iBAG and non-INT models. For a fair comparison, we use a Bayesian approach to obtain estimations for all three models using double-exponential (lasso) priors. We construct the priors for the iBAG*_unified_* model as stated in Section 2.3. The priors for the non-INT model are the same as those for the iBAG*_unified_* model, except for setting 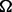 to be 0. The priors for the ADD model are the same as those in the non-INT model, except for the priors of the methylation effects, which are set in a manner similarly to that of the priors for gene expression effects in the non-INT model. To check the convergence of the iBAG*_unified_* model, we run two MCMC chains with different starting values. As seen in the trace plots and the plots based on Gelman and Rubin’s convergence diagnostic statistics, for the important parameters in the iBAG*_unified_* model (Supplementary Fig. S5), the results show that the iBAG*_unified_* model converges after ∼2000 iterations.

To compare the performance of the three models, we obtain the predicted values for the test dataset using the mean estimations of the parameters from the posterior samples for all three models. We use the concordance index (C-index) to evaluate the prediction performance for the different models. The C-index can be expressed as 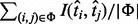, where 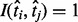 for 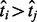 and = 0 otherwise, 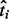 is the estimated survival time for patient *i*, and Φ is the set that consists of all pairs of *i, j* such that survival time *t_i_* > *t_j_*. This measure has been shown to be effective in comparing prediction performances among different models for right-censored data (Bonato *et al.*, 2011; Harrel *et al.*, 2001; van Wieringen *et al.*, 2009). We calculated C-indexes for the fitted values in the training dataset and the predicted values in the test dataset for all three models. The results are summarized in Table 1. The C-indexes for the iBAG*_unified_* model are the highest for both the training (0.80) and test datasets (0.76). The C-indexes for the non-INT model are the lowest for both training (0.70) and test datasets (0.73). Although the improvements by integrating the methylation data are limited (all 95% CIs of the C-index overlapped for the three models), the iBAG*_unified_* model has the best performance in both model fitting and model prediction.

**Table 1.** C-indexes for the three models in the training and test datasets

	non-INT model	ADD model	iBAG*_unified_* model
Training data	0.73 (0.02)	0.77 (0.03)	0.80 (0.03)
Test data	0.70 (0.03)	0.75 (0.02)	0.76 (0.03)

**Fig. 4.**
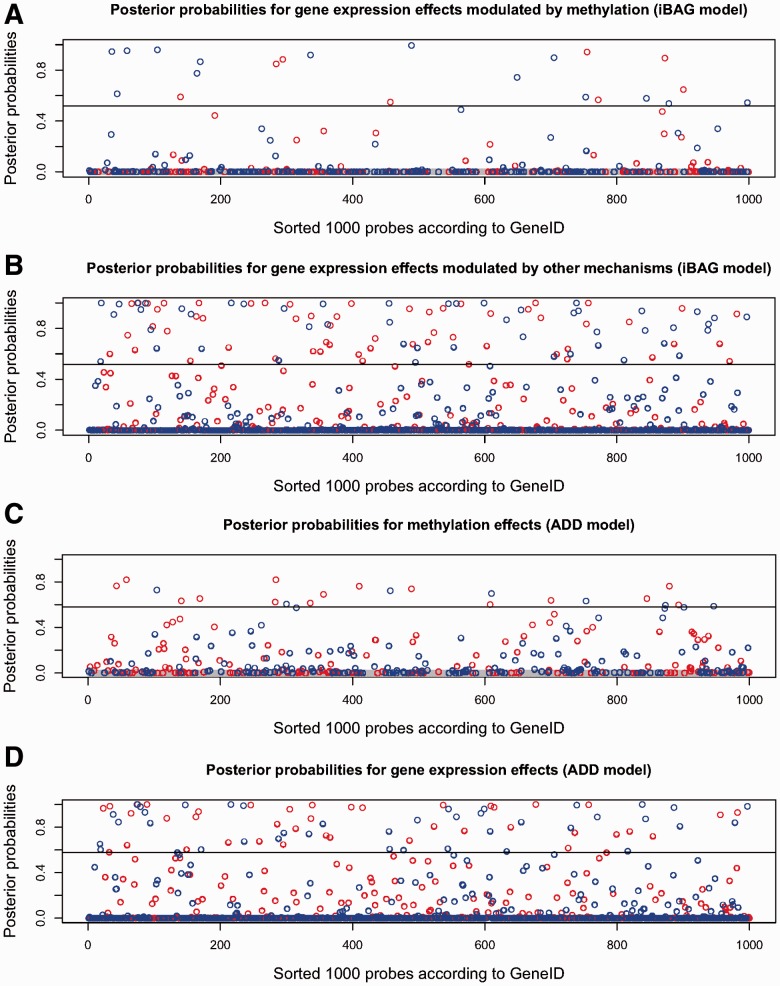
Posterior probabilities for gene expression effects by the iBAG*_unified_* model (panel A for effects modulated by methylation and panel B for effects modulated by other mechanisms), by the ADD model (panel C for effects identified by methylation and panel D for effects identified by gene expression). Blue dot: Negative effect (higher expression indicates shorter survival); Red dot: Positive effect (higher expression indicates longer survival); Black horizontal line: corresponding cutoff for posterior probabilities at FDR = 0.2

For the two models performing relatively better in prediction (iBAG*_unified_* model and ADD model), we use Gibbs sampling to obtain posterior samples for the parameters and apply the method described in Section 3 to obtain the posterior probabilities for the different types of gene expression effects. The Bayesian posterior probabilities obtained by the iBAG model for the type *M* and type 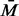 effects are summarized in Figure 4, panels A and B, respectively. The Bayesian posterior probabilities of the methylation effects and gene expression effects by the ADD model are summarized in Figure 4, panels C and D, respectively.

Applying the iBAG*_unified_* model to the GBM training dataset, we identify 136 genes (of the total 348 genes with methylation information) as significantly modulated by at least one methylation feature. These genes are listed in Supplementary Table S3. Of 136 genes, 102 genes (76%) have a negative estimation for methylation effects. This result reflects the biologic action of methylation, which usually represses gene expression. At FDR = 0.2 (corresponding posterior probability cutoff = 0.517), we obtain 22 genes with non-zero type *M* effects (effects modulated by methylation) on patient survival using the iBAG*_unified_* model. These genes are listed in the top box of Table 2. We use an asterisk to show the genes significantly modulated by methylation, as identified by the iBAG*_unified_* model (within the list provided in Supplementary Table S1). We use a boldface font to show the genes positively associated with patient survival (higher expression of the gene indicates longer survival time), and a regular font to show the genes negatively associated with patient survival (higher expression of the gene indicate shorter survival time). In addition, we identify 107 genes with nonzero type 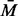 effects on survival (summarized in the lower box in Table 2). CNGA3 is the only gene that overlaps between the 22 genes with type *M* effects and the 107 genes with type 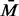 effects, which means that CNGA3 is found to have effects modulated by both methylation and other mechanisms.

**Table 2.** Genes with significant gene expression effects obtained by iBAG*_unified_* model at FDR = 0.05 sorted according to their GeneIDs

Type *M* genes	SPON2, CAP2*, POLR3C, CNGA3*, DPP4, GPR116, FKBP1A, SARM1*, RNF115*, HOXA1*, PCP4*, CYB5R2, RBBP4, SMURF2, TK1, C1QA*, UFD1L*,C2orf44*, SF3B5*,CASP4, CBFB*, MVP* LPCAT3, TRIB1, **PEMT**, TAB1, DCTN2, **FARS2**, **RPP40**, **PNPLA6**, OS9, **SLC27A5**, TMEM115, POLI, **NXPH3**, ADCY8, C16orf42*, CLTC, **STX2**, **SEP10**, **E2F4**, CNGA3*, **AIM1**, CSTA*, FCER1G*, FHIT*, **ZBTB1**, **FRAT2**, NPTXR, PISD, **CCDC19**, FAM50B*, ZNF544*, DKK3*, SREBF1, **GRIK5**, **GSTM3**, MNX1*, HSPA1A*, **IGBP1**, IL10RB, **INPPL1**, IPW, **ITPR2**, **KARS**, **LRP3**, **MAP3K10**, NCF2*, ATIC*, **ACO1**
Type 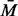 genes	**PABPC3**, MRTO4, VPS28, PDE8A*, **ENPP2**, **WBP11,** PFDN2*, PHKG1, **POLR2H**, **RC3H2**, **NDE1**, **FBXO34**, ARHGEF10L, **C12orf35**, PPP2R2A, ADI1, GIMAP5*, **AMBRA1**, BIN3, **UBFD1**, **BEX4**, EPB41L5, RGS3*, ELOVL5, RPE*, **RPS4X**, CFB*, PLEK*, **PORCN**, SP3*, SP100*, GNS, STAT6*, SURF2, TACC1, **HOXC4**, **TLE1**, **TOP1**, UBE2V2, VDAC3, SLC39A7, KIAA1012, **ADIPOR2**, SLC24A6, ZNF430, **NPRL3**, SH3BGRL3*, ZNF528, MT4*, CSDA, **RUVBL1**, HERC2, DIRAS3*, EIF1AY, VAPB, **RPL23**, **SNCAIP**, **KIAA0141**, HS3ST2

Type *M* effects: effects modulated by methylation; type 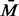 effects: effects modulated by other mechanisms; asterisk: genes significantly modulated by methylation; genes in bold font: Genes positively associated with patient survival; Genes in regular font: Genes negatively associated with patient survival.

Using the ADD model with the same FDR = 0.2 (corresponding posterior probability cutoff = 0.579), we obtain 22 genes that have significant methylation effects and 78 genes that have significant gene expression effects on patient survival. By comparing the gene lists derived by the iBAG and ADD models using Venn diagrams (Supplementary Figs S4.1 and S4.2), we observe that 59 of 78 genes with significant gene expression effects obtained by the ADD model overlap with the genes with non-zero type 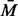 effects obtained by the iBAG*_unified_* model, and have a directional association with survival time (both are positive or negative). There are 12 common genes when comparing the genes with significant methylation effects by the ADD model and the genes with non-zero type *M* effects (effects modulated by methylation). Among the 10 genes with non-zero type *M* effects obtained only by the iBAG*_unified_* model, five genes (SARMS1, C1QA, UFD1L, CBFB and MVP) are found to be significantly modulated by methylation (see Supplementary Table S1). However, for the 10 genes with significant methylation effects obtained only by the ADD model, only two of them (ANK3 and IL11RA) are found to be significantly modulated by methylation (see Supplementary Table S1). This indicates that for the other eight genes obtained only by the ADD model, they are shown to have significant methylation effects on survival, but their gene expression levels are not changed. This result does not seem to conform to our belief that methylation affects patient survival by depressing the gene expression. The advantage of the iBAG*_unified_* model is that it can identify the genes with effects modulated by methylation, and thus the results are more biologically interpretable.

Of the 22 genes identified by effects modulated by methylation, 14 are negatively associated with survival, whereas eight genes are positively associated with survival. Functional analysis with the database for annotation, visualization and integrated discovery (DAVID, Dennis *et al.*, 2003) revealed that some of the genes that are negatively associated with survival are regulators of transcription (SMURF2, HOXA1, RBBP4 and POLR3C) and code for plasma membrane (CAP2, GPR116, SMURF2). Detailed results and additional discussions can be found in Supplementary Table S5.1. This gene set related with negative survival is enriched for Gene Ontology terms, cell morphogenesis and neuron differentiation, suggesting a probable role in the genesis of brain tumors. On the other hand, the effects of genes associated with positive survival are mostly intra-cellular and are related to immune systems processes (C1QA, CBFB, DPP4 and SARM1), suggesting a likely function in tumor suppression (see Supplementary Table S5.2). Although no GBM studies have so far identified these 22 genes as important biomarkers of survival, two of the genes in this list are associated with other types of glioma—MVP was found to be overexpressed in ganglio-gliomas (Aronica *et al.*, 2003). Moreover, most of the genes in this list have important biological functions in other types of cancer. For example, HOXA1 stimulates oncogenesis through the MAPK signaling pathway and the transcription factors STAT3 and STAT5B in mammary epithelial cells (Mohankumar *et al.*, 2008). Also, CpG islands of HOXA1 are significantly hypermethylated in lung cancer (Selamat *et al.*, 2011), breast cancer (Park *et al.*, 2011) and gastric carcinoma (Kang *et al.*, 2008).

## 6 DISCUSSION

In this article, we introduce an innovative model, iBAG, to integrate two different platforms of *omics* data and estimate their associations with clinical outcome. Different from most existing integration approaches, which focus on either finding biological relationships among different platforms or predicting patient prognosis, our iBAG model involves a hierarchical structure, which simultaneously estimates biological mechanisms and uses this information to find significant prognostic genes. Our simulation study shows that the iBAG model can simultaneously increase the power and decrease the FDR in detecting clinically relevant genes, especially for genes with expression effects modulated only by methylation. Moreover, we can categorize all clinically relevant genes into three groups according to different biological mechanisms: genes with expression effects modulated only by methylation, genes with expression effects modulated only by other mechanisms and genes with expression effects modulated by both methylation and other mechanisms. We apply the iBAG model to integrate methylation data and gene expression data from TCGA’s GBM dataset. The results show that the iBAG model outperforms the model based on data from a single layer of biological information in both determining genes important to survival and model fitting.

The main goal of the iBAG model is to (i) identify more disease-associated genes, and (ii) achieve better predictive power, by treating gene expression as the downstream event that is regulated by different mechanisms (e.g. methylation, copy number and microRNA). We choose to treat the gene expression as a downstream event regulated by different mechanisms so that the iBAG model can help us identify more disease-associated genes. There are several reasons underlying this choice. First, acknowledging that a gene’s expression can be modulated by different mechanisms (e.g. methylation, copy number and microRNA), even if these mechanisms do not have a direct effect on survival, we can still identify genes whose modulated expressions potentially impact survival. Second, as the measure of gene expression from microarray technology is usually noisy, iBAG can effectively identify, which part of the gene expression is actually modulated by various factors from other platforms, thus denoising the expression to find prognostic genes. In addition, as shown by our analysis of the GBM data, if we simply use methylation information additively to gene expression to estimate the methylation effects on patient survival, we find that many methylation effects related to survival do not significantly change the gene expression levels. The iBAG model can help us eliminate these genes and obtain results that are more biologically interpretable.

As our main goal is to identify important genes associated with patient survival, we assume that the methylation effect on gene expression and the gene expression effect on patient survival are all linear and independent. By making this assumption, the conditional posterior distributions are in closed forms, which save us on computation cost. However, this assumption may not reflect the true biological process; therefore, if the main interest is to make predictions about clinical outcome, then more general forms of functions (e.g. non-parametric functions) may need to be considered. In our study, we focus on finding purely associational relationships between genes and patients’ survival times. Independent functional experiments and datasets are needed to validate any causal relationships or implications. In addition, although our implementation is Bayesian, the fundamental idea of the integrative hierarchical modeling can be applied using frequentist approaches as well. Although we illustrate the integration of only two platforms at a time, integrating three or more platforms can also be done by following a similar framework. This will require a deeper understanding of the fundamental biological relationships among different data platforms. We leave these tasks for future consideration. The iBAG model provides a useful and intuitive framework for integrating multiple platforms to improve diagnosis and prognosis in cancer. A freely available R software for the iBAG model is available under the ‘software’ link at: http://odin.mdacc.tmc.edu/ ∼vbaladan/.

## Supplementary Material

Supplementary Data
