# Supplementary material for: iBAG: integrative Bayesian analysis of high-dimensional multiplatform genomics data
Source: Bioinformatics. 2012 Nov 9;29(2):149–59. doi: 10.1093/bioinformatics/bts655 (PMC3546799; doi:10.1093/bioinformatics/bts655)
Supplement: Supplementary Data [file supp_29_2_149__index.html]

Integrative Bayesian Analysis of High-dimensional Multi-platform Genomics Data — iBAG: integrative Bayesian analysis of high-dimensional multiplatform genomics data — Supplementary Data 

# iBAG: integrative Bayesian analysis of high-dimensional multiplatform genomics data

## Supplementary Data

files

**Files in this Data Supplement:**

- Supplementary Data - pdf file
