# Supplementary material for: iBAG: integrative Bayesian analysis of high-dimensional multiplatform genomics data
Source: Bioinformatics. 2012 Nov 9;29(2):149–59. doi: 10.1093/bioinformatics/bts655 (PMC3546799; doi:10.1093/bioinformatics/bts655)
Supplement: Supplementary Data [file supp_bts655_supplementary_materials_iBAG_FINAL_online.pdf]

Supplementary Materials for Integrative Bayesian Analysis of High-Dimensional Multi-platform Genomics Data

by

Wenting Wang, Veerabhadran Baladandayuthapani, Jeffrey S. Morris, Bradley M. Broom, Ganiraju C. Manyam, and Kim-Anh Do

This supplementary material is structured as follows. In Sections S1 and S2, we present detailed information about the full conditional posterior distributions of the iBAG model and the associated posterior sampling schemes. In Section S3, we present the additional simulation results to supplement those presented in the main manuscript. In Section S4, we describe the pre-processing procedures for The Cancer Genome Atlas (TCGA) glioblastoma (GBM) study, and we present some additional results obtained by the iBAG model.

S1: Full conditional posterior distributions of the iBAG model for continuous responses

We first present the directed acyclic graph (DAG) representation of the iBAG model in Figure S1. The observed data are represented by square nodes (e.g., the methylation levels, $\mathbf{M}$, the gene expression levels, $\boldsymbol{g}_k$s, and the patient clinical outcome $\boldsymbol{Y}$), and the parameters are represented by circular nodes (e.g., the methylation effects, Ω, and the gene expression effects, $\boldsymbol{\gamma}^M$ and $\boldsymbol{\gamma}^{\bar{M}}$.). The nodes within the grey rectangle have K replicates. The arrows in the figure show the dependency structures for different nodes.

We provide the detailed, full confidential likelihood of the iBAG model that is introduced in Section 2.3 of the main article. All of the notations used here follow those introduced in the main article.

Based on the results obtained from Andrews and Mallows (1974), the double exponential distribution can be written as a mixture of a normal distribution with an exponential density. Specifically, the prior for the type M effect for the kth gene ($[\boldsymbol{\gamma}_k^M|\lambda^M,\sigma]$) can be rewritten as:

$$\frac{\lambda^M}{2\sigma}\exp(-\lambda^M|\gamma_k^M|) = \int_0^\infty \frac{1}{\sqrt{2\pi x_k^M}}\exp\left(-\frac{(\gamma_k^M)^2}{2x_k^M}\right)\frac{(\lambda^M)^2}{2\sigma^2}\exp\left(-\frac{(\lambda^M)^2}{2\sigma^2}x_k^M\right)dx_k^M, \tag{S1}$$

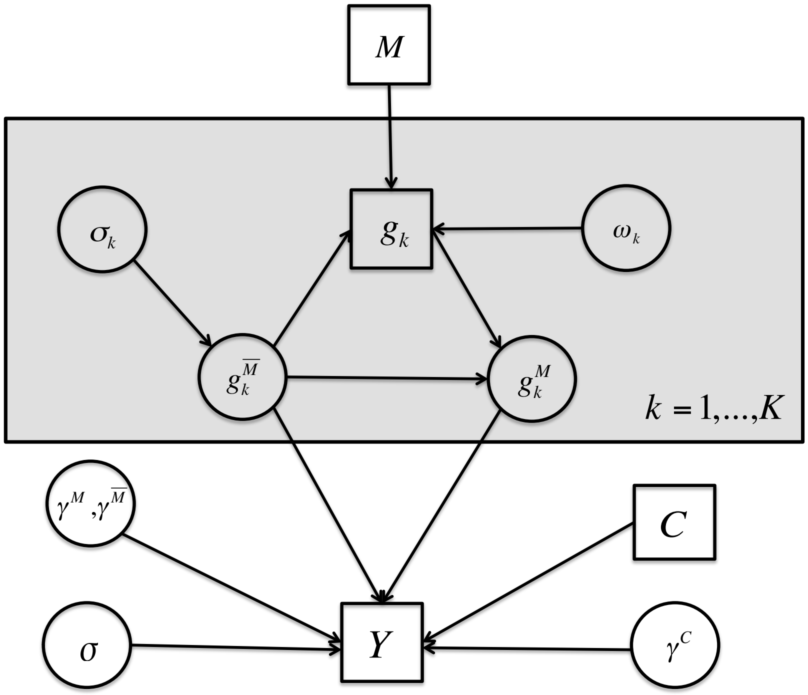

Figure S1: Directed acyclic graph representation of the iBAG model

for $k = 1, \ldots, K$. And similarly, the prior for the type $\bar{M}$ effect for the kth gene ($[\boldsymbol{\gamma}_k^{\bar{M}}|\lambda^{\bar{M}}, \sigma]$) can be rewritten as:

$$\frac{\lambda^{\bar{M}}}{2\sigma}\exp(-\lambda^{\bar{M}}|\gamma_k^{\bar{M}}|) = \int_0^\infty \frac{1}{\sqrt{2\pi x_k^{\bar{M}}}}\exp\left(-\frac{(\gamma_k^{\bar{M}})^2}{2x_k^{\bar{M}}}\right)\frac{(\lambda^{\bar{M}})^2}{2\sigma^2}\exp\left(-\frac{(\lambda^{\bar{M}})^2}{2\sigma^2}x_k^{\bar{M}}\right)dx_k^{\bar{M}}, \tag{S2}$$

for $k = 1, \ldots, K$.

Let $(\tau_k^M)^2 = x_k^M/\sigma^2$ and $(\tau_k^{\bar{M}})^2 = x_k^{\bar{M}}/\sigma^2$. Based on equation S1 and equation S2, the priors for $\boldsymbol{\gamma}^M$ and $\boldsymbol{\gamma}^{\bar{M}}$ can be rewritten as

$$[\boldsymbol{\gamma}_k^M|\sigma^2, \mathbf{D}_M] \sim MN_K\left(0, \sigma^2\mathbf{D}_M\right),\ [\boldsymbol{\gamma}_k^{\bar{M}}|\sigma^2, \mathbf{D}_{\bar{M}}] \sim MN_K\left(0, \sigma^2\mathbf{D}_{\bar{M}}\right),$$

where

$$\mathbf{D}_M = \text{Diag}\left((\tau_1^M)^2, \ldots, (\tau_K^M)^2\right),\ \mathbf{D}_{\bar{M}} = \text{Diag}\left((\tau_1^{\bar{M}})^2, \ldots, (\tau_K^{\bar{M}})^2\right),$$

and

$$[(\tau_1^M)^2, \ldots, (\tau_K^M)^2] \sim \prod_{k=1}^{K} \frac{(\lambda^M)^2}{2} \exp\{-(\lambda^M \tau_k^M)^2/2\} d(\tau_k^M)^2,$$

$$[(\tau_1^{\bar{M}})^2, \ldots, (\tau_K^{\bar{M}})^2] \sim \prod_{k=1}^{K} \frac{(\lambda^{\bar{M}})^2}{2} \exp\{-(\lambda^{\bar{M}} \tau_k^{\bar{M}})^2/2\} d(\tau_k^{\bar{M}})^2.$$

The full conditional distributions for the parameters in the *mechanistic model* can be expressed as follows.

- $[\omega_{j_0,k_0}|\text{others }]$, the methylation level of the j_0th methylation effect on the mRNA level of the k_0th gene:

$$N\left((\sigma^{-2}(\boldsymbol{g}^{k_0})'\boldsymbol{g}^{k_0} + \sigma_{k_0}^{-2}\boldsymbol{m}_{j_0}'\boldsymbol{m}_{j_0} + 10^{-6})^{-1}(\sigma^{-2}(\boldsymbol{g}^{k_0})'\boldsymbol{Y}^{k_0} + \sigma_{k_0}^{-2}\boldsymbol{m}_{j_0}'\boldsymbol{g}_{k_0})\ ,\right.$$
$$\left.(\sigma^{-2}(\boldsymbol{g}^{k_0})'\boldsymbol{g}^{k_0} + \sigma_{k_0}^{-2}\boldsymbol{m}_{j_0}'\boldsymbol{m}_{j_0} + 10^{-6})^{-1}\right),$$

  for $k_0 = 1, \ldots, K$, and $\boldsymbol{m}_{j_0}$ within the promoter of the k_0th gene, where

$$\boldsymbol{g}^{k_0} = (\boldsymbol{\gamma}_{k_0}^M - \boldsymbol{\gamma}_{k_0}^{\bar{M}})\boldsymbol{m}_{j_0}, \text{ and } \boldsymbol{Y}^{k_0} = \boldsymbol{Y} - \mathbf{G}\boldsymbol{\gamma}^{\bar{M}} - \sum_{k \neq k_0} \left\{\omega_{jk}(\gamma_k^M - \gamma_k^{\bar{M}})\boldsymbol{m}_{j_0}\right\}.$$

- $[\sigma_{k_0}^2|\text{others}]$, the random error modeling the part of the expression of the k_0th gene that is not explained by methylation:

$$\text{Inverse Gamma}\ \left(\frac{N-1+J}{2}, \frac{1}{2}(\boldsymbol{g}_{k_0} - \omega_{k_0}\boldsymbol{m}_{k_0})'(\boldsymbol{g}_{k_0} - \omega_{k_0}\boldsymbol{m}_{k_0}) + \frac{10^{-6}}{2}\omega_{k_0}^2\right).$$

The full conditional distributions for the parameters in the *clinical model* can be expressed as follows.

- $[\boldsymbol{\gamma}^C|\text{ others}]$, the effects of clinical factors (e.g., tumor stage) on clinical outcome:

$$MN_L\left(\{\sigma^{-2}\mathbf{C}'\mathbf{C} + 10^{-6}\mathbf{I}\}^{-1}\mathbf{C}'(\boldsymbol{Y} - \mathbf{G}^{\bar{M}}\boldsymbol{\gamma}^{\bar{M}} - \mathbf{G}^M\boldsymbol{\gamma}^M), \{\sigma^{-2}\mathbf{C}'\mathbf{C} + 10^{-6}\}^{-1}\right).$$

- $[\boldsymbol{\gamma}^M|\text{ others}]$, the effects of gene expression modulated by methylation:

$$MN_K\left(\{(\mathbf{G}^M)'\mathbf{G}^M + \mathbf{D}_M^{-1}\}^{-1}(\mathbf{G}^M)'\boldsymbol{Y}^M, \sigma^2\{(\mathbf{G}^M)'\mathbf{G}^M + \mathbf{D}_M^{-1}\}^{-1}\right).$$

- $[\boldsymbol{\gamma}^{\bar{M}}|\text{ others}]$, the effects of gene expression modulated by other sources of genomic changes, follows

$$MN_K\left(\{(\mathbf{G}^{\bar{M}})'\mathbf{G}^{\bar{M}} + \mathbf{D}_{\bar{M}}^{-1}\}^{-1}(\mathbf{G}^{\bar{M}})'\boldsymbol{Y}^{\bar{M}}, \sigma^2\{(\mathbf{G}^{\bar{M}})'\mathbf{G}^{\bar{M}} + \mathbf{D}_{\bar{M}}^{-1}\}^{-1}\right).$$

- $[\sigma^2_{k_0}|$ others], the standard deviation for the error term that accounts for variation not accounted for by the observed genomic and clinical factors:

Inverse Gamma
$$\left(\frac{N-1+2K}{2}, \frac{1}{2}(\boldsymbol{Y}^M + \boldsymbol{Y}^{\bar{M}} - \boldsymbol{Y})'(\boldsymbol{Y}^M + \boldsymbol{Y}^{\bar{M}} - \boldsymbol{Y}) + \frac{\lambda^M}{2}(\boldsymbol{\gamma}^M)'\mathbf{D}^{-1}_M\boldsymbol{\gamma}^M + \frac{\lambda^{\bar{M}}}{2}(\boldsymbol{\gamma}^{\bar{M}})'\mathbf{D}^{-1}_{\bar{M}}\boldsymbol{\gamma}^{\bar{M}}\right),$$
where $\boldsymbol{Y}^{\boldsymbol{M}} = \boldsymbol{Y} - \mathbf{G}^{\bar{M}}\boldsymbol{\gamma}^{\bar{M}}$ and $\boldsymbol{Y}^{\bar{M}} = \boldsymbol{Y} - \mathbf{G}^M\boldsymbol{\gamma}^M$.

- $[(\tau_k^M)^{-2} = \eta_k^M|$ others]:

$$\text{Inverse Gaussian}\left(\lambda^M\sigma_0/|\gamma_k^M|, (\lambda^M)^2\right)\mathbf{I}(\eta_k^M > 0).$$

- $[(\lambda^{\bar{M}})^2|$ others]:

$$\text{Inverse Gaussian}\left(\lambda^{\bar{M}}\sigma_0/|\gamma_k^{\bar{M}}|, (\lambda^{\bar{M}})^2\right)\mathbf{I}(\eta_k^{\bar{M}} > 0).$$

The full conditional distributions for the *shrinkage parameters* can be expressed as follows.

- $[(\lambda^M)^2|$ others], the common shrinkage parameter for the gene expression effects modulated by methylation:

$$\text{Gamma}\ \left(K + \alpha^M, \sum\frac{1}{\eta_k^M} + \xi^M\right).$$

- $[(\tau_k^{\bar{M}})^{-2} = \eta_k^{\bar{M}}|$ others], the common shrinkage parameter for the gene expression effects modulated by methylation:

$$\text{Gamma}\ \left(K + \alpha^{\bar{M}}, \sum\frac{1}{\eta_k^{\bar{M}}} + \xi^{\bar{M}}\right).$$

Hence, the posterior samples for the parameters in the iBAG model for continuous responses can be drawn by Gibbs sampling.

S2: Sampling schemes for the iBAG model for discrete/survival responses

In Section 2.4 of the main article, we introduced a continuous latent variable $\boldsymbol{Z}$ as a data augmentation step for our MCMC schemes for discrete and survival responses. For a binary response variable, the relationship between $\boldsymbol{Z}$ and the response variable $\boldsymbol{Y}$ can be expressed as

$$Y_n = \begin{cases} 1 & \text{if } Z_n > 0 \\ 0 & \text{otherwise} \end{cases} \quad \text{for } n = 1, \ldots, N. \tag{S2}$$

For a right-censored response variable, the relationship between $\boldsymbol{Z}$ and the response variable $\boldsymbol{Y}$ can be expressed as

$$\begin{cases} \log(t_n) = Z_n & \text{if } \delta_n = 1 \\ \log(t_n) > Z_n & \text{if } \delta_n = 0 \end{cases} \quad \text{for } n = 1, \ldots, N. \tag{S3}$$

Conditionally on $\boldsymbol{Z}$, iBAG models for both discrete and right-censored responses are the same as that for continuous responses.

The drawing scheme for parameters of the iBAG model with binary or censored outcomes has the following steps:

1) Update $(\boldsymbol{\gamma}^M, \boldsymbol{\gamma}^{\bar{M}}, \boldsymbol{\Omega})$ using Gibbs sampling.

2) Update the variance for the random error term $(\sigma,\ \sigma_1, \ldots, \sigma_K)|(\boldsymbol{\gamma}^M, \boldsymbol{\gamma}^{\bar{M}}, \boldsymbol{\Omega})$, using Gibbs sampling.

3) Update Z according to equation (S2) if patient clinical outcomes are binary or equation (S3) if patient clinical outcomes are censored.

S3: Additional simulation results

S3.1: ROC analysis

In Section 4 of the main paper, we generate 12 different scenarios based on different combinations of total number of gene expression features, and the correlations between methylation and gene expression features. We fit four models, $\text{iBAG}_{\text{unified}}$, $\text{iBAG}_{\text{2-stage}}$, single gene (SG), and non-integrative (nonINT) model, for all the simulated datasets. In this section, we show the receiver operating characteristic (ROC) curves in identifying the true effects of gene expressions and calculate the areas under these ROC curves (AUCs) for all 12 simulated scenarios in Figures S2.1 to S2.3. Figure S2.1 summarizes the true positive rate (TPR) versus the false positive rate (FPR) for discovering genes with only type M effects (effects modulated only by methylation). Figure S2.2 summarizes the TPR versus the FPR for discovering genes with only type $\bar{M}$ effects (effects modulated only by other mechanisms). Figure S2.3 summarizes the TPR versus the FPR for discovering genes with type $M + \bar{M}$ effects (effects modulated by both methylation and the other mechanisms). The different line colors in these figures represent different models; different line types represent varying correlations between gene expression and methylation features (see the figure legends). The AUCs of the corresponding ROC curves are enclosed within parentheses in the legends.

In Figure S2.1, since only the first 200 genes in the gene expression dataset are modulated by methylation, and genes 181 to 200 have effects modulated by both methylation and other mechanisms, we include only the first 180 genes when we calculate the TPR and the FPR. As expected, the nonINT model performs the worst of all three models, followed by the $\text{iBAG}_{\text{2-stage}}$ model and the SG model. The proposed $\text{iBAG}_{\text{unified}}$ model performs the best of all three models.

All of the genes are included when we calculated the TPR and the FPR shown in Figure S2.2, except genes 1 to 20, which have effects modulated by only methylation, and genes 181 to 200, which have effects modulated by both methylation and other mechanisms. This figure shows that the SG model performs the worst in discovering the genes with type $\bar{M}$ effects. The nonINT model performs slightly better than the $\text{iBAG}_{\text{2-stage}}$ model in discovering this group of genes. The proposed $\text{iBAG}_{\text{unified}}$ model performs the best of all three models.

All of the genes are included when we calculate the TPR and the FPR shown in Figure S2.3, except genes 1 to 20, which have effects modulated only by methylation, and genes 201 to 220, which have effects modulated only by other mechanisms. This figure shows that the SG model performs the worst in discovering the genes with effects modulated by both methylation and other mechanisms. The $\text{iBAG}_{\text{2-stage}}$ model performs as well as the nonINT model in discovering the genes in this group of genes. The proposed $\text{iBAG}_{\text{unified}}$ model performs the best of all three models.

S3.2: Model performance and consistency

For the situation that best mimics our real data scenario ($K = 1000$ and $\rho = -0.6$), we ranked the performance of four different models in identifying different sets of genes based on their AUC values. Table S1 shows that our proposed $\text{iBAG}_{\text{unified}}$ model performs the best of all three models in discovering all three group of genes. The $\text{iBAG}_{\text{2-stage}}$ model ranks second except for in one case. The SG model and the nonINT model have the worst performances.

In addition, we evaluate the consistency of the $\text{iBAG}_{\text{unified}}$ model, both in terms of model selection and estimation. We generate 100 datasets for the simulation scenario in which the total number of genes (K) is 1000 and the methylation-gene expression correlation is equal to -0.6. To evaluate the consistency of the $\text{iBAG}_{\text{unified}}$ model in variable (gene) selection, we summarize the posterior probabilities for the genes with nonzero and zero effects using barplots with standard errors across the 100 datasets (see panels A and B in Figure S3). In addition, we calculate the AUCs for identifying genes with nonzero effects for each dataset. The barplots of the AUCs from 100 datasets are shown

Table S1: Rank of performance for identifying genes by four different models based on the area under the ROC curves

	Gene Type		
Model	Type M	Type $\bar{M}$	Type $M+\bar{M}$
nonINT model	4	2	3
SG model	3	4	2
iBAG$_{\text{2-stage}}$ model	2	3	4
iBAG$_{\text{unified}}$ model	1	1	1

in Figure S3, panel C. Panels A and B show that for the 100 simulated datasets, the genes with nonzero gene expression effects consistently have higher posterior probabilities than the genes with zero gene expression effects modulated both by methylation (γ^M) and other genomic changes ($\gamma^{\bar{M}}$). This leads to higher AUCs in selecting genes that are important (with nonzero effects) to clinical outcomes. As shown in panel C, the AUCs based on the iBAG$_{\text{unified}}$ model are about 0.8, with very small standard errors in identifying both gene effects modulated by methylation and gene effects modulated by other genomic changes. Hence, from Figure S3, we conclude that the iBAG$_{\text{unified}}$ model demonstrates good (empirical) consistency in selecting relevant variable (genes).

The mean biases for some of the regression and variance parameters over the 100 datasets, along with the standard errors, are summarized in Table S2. We observe some bias in our parameter estimates, with the bias being slightly higher for true (non-zero) effects than for zero effects. Although we observe good (empirical) consistency in our model selection, biases are observed in our parameter estimates are for two reasons. First, our construction of the double exponential prior has a single (global) shrinkage parameter for all the regression coefficients (large and small), which may tend to over-shrink large effects and under-shrink the small effects. This problem can be alleviated by assuming adaptive versions of the lasso prior such as normal-gamma-based priors (Griffin and Brown, 2010, 2011), which allows for additional flexibility, but at the cost of additional computation. Second, the consistency of the estimations of regression coefficients in the lasso model has been proven by Knight and Fu (2000) as the sample size goes to infinity. However, in our case, since the sample size (number of patients, N) is much smaller than the total number of predictors (total number of genes, K), this may lead to bias in our parameter estimates.

Table S2: Mean biases for parameters in the iBAG$_{\text{unified}}$ model, estimated from 100 simulation datasets (total number of genes: $K = 1000$, methylation-gene expression correlation: $\rho = -0.6$)

Parameter	γ^M		$\gamma^{\bar{M}}$		β	σ_m	σ
True value	$\gamma^M = 1$	$\gamma^M = 0$	$\gamma^{\bar{M}} = 1$	$\gamma^{\bar{M}} = 0$	$\beta = -1.5$	$\sigma_m = 0.44$	$\sigma = 0.1$
Mean of bias	0.63	0.09	0.51	0.08	0.13	0.02	0.18
SE of bias	0.05	0.012	0.03	0.007	0.009	0.005	0.015

S4: Additional results for TCGA GBM data analysis

In this section, we explain the pre-preprocessing steps for the TCGA GBM dataset before applying the iBAG$_{\text{unified}}$ model, and provide additional analysis results.

S4.1 Pre-processing procedures

Methylation data: The DNA methylation information is obtained by using the Illumina Human methylation 27 BeadChip. Level 3 data are downloaded directly from the TCGA website (http://tcga-data.nci.nih.gov/tcga/tcgaHome2.jsp). The downloaded data contain the measures of normalized unmethylated (U) and methylated (M) levels for 27,578 highly informative CpG sites per sample at single-nucleotide resolution. We calculate the beta values for each site, which equal $M/(U + M)$. The beta value is a number between zero and one that measures the percentage of DNA methylation on a site. Next, we remove the methylation sites from our analysis if fewer than 5% or more than 95% of samples have beta values less than 0.5. The purpose of this step is to ensure that the beta values for the selected probes vary by patient. After this step, we have 6890 sites left.

Gene expression data: The gene expression data are obtained by using Affymetrix Human Genome U133A Array, which consists of the expression level of 12,000 well-characterized human genes. Level 2 data are downloaded from the TCGA website. The data are normalized globally using BrainArray CDF and the RMA normalization method. This normalization process generates one unique expression level for each human gene. We then remove the underexpressed genes according to the following rule: Patients are divided into a short survival group and a long survival group using 2 years as the cutoff point. A gene is defined to be underexpressed and removed if the means of the normalized expression for both groups are less than 5. This leaves us with 7785 genes.

Annotate methylation features to genes: For each of the 7785 genes, we can obtain the per-

centage of methylation for all the sites within its promoter using the following steps. (1) For each gene expression, find the GeneID for this gene using the annotation file for U133A Array. (2) For each gene, determine the corresponding coordinate for the transcription start site (TSS) of this gene by using the annotation file for methylation. (3) From the 6890 sites that pass the methylation filter, obtain all sites within −5kb to 0.5kb from the TSS of each gene (gene promoter) according to the methylation map information.

Univariate iBAG$_{\textbf{2-stage}}$ model: In our analysis, we include age as one of the predictors because it has been shown to be an important predictor for GBM patient survival. Although we have measures from 7785 genes, in our analysis, we focus on only the top 1000 genes chosen using the following steps. First, for each of the 7785 genes, a linear regression model is fitted using the beta values from its promoter as explanatory variables and the gene expression as the response variable. Thus we can obtain a fitted value and a residual for each gene. If there is no methylation information measured within the promoter of a gene, the fitted value of the gene expression probe is set to zero. Second, we fit a univariate AFT model for the fitted value and the residual for each gene expression probe separately after adjusting for age. The p-values for both fitted values and residuals in the univariate AFT models are combined, and the 1000 genes with the smallest p-values are identified. Out of the 1000 genes, 348 genes have had the methylation level measured within their promoters.

S4.2 Additional analysis results

As explained in the main paper, we fit three models to the processed GBM dataset. The first model is the nonINT model, with only gene expression information as explanatory variables. The second model is the additive (ADD) model, with both gene expression and methylation information as explanatory variables and assuming their effects on patients' survival times are additive. The third model is the iBAG$_{\text{unified}}$ model for censored outcomes, which integrates both gene expression and methylation information in a hierarchical manner.

In Table S3, we provide a list of 136 genes that are identified as significantly modulated by at least one methylation feature using the iBAG$_{\text{unified}}$ model, based on 95% credible intervals. In Tables S4.1 and S4.2, we provide a summary of the number of significant prognostic genes for the iBAG$_{\text{unified}}$ and ADD models, respectively, along with their type of effects. In addition, we use a Venn diagram to compare the gene lists derived by the iBAG$_{\text{unified}}$ and ADD models (Figure S4). We observe that 59 out of 78 genes with significant gene expression effects obtained by the ADD

model overlap with the genes with nonzero type $\bar{M}$ effects obtained by the $\text{iBAG}_{\text{unified}}$ model, and affect survival time in the same direction (both are positive or negative). There are 12 common genes identified from the comparison of genes with significant methylation effects (using the ADD model) to genes with nonzero type M effects (effects modulated by methylation).

Of the 22 genes identified by effects modulated by methylation, 14 are negatively associated with survival, while 8 genes are positively associated with survival. Functional analysis with the database for annotation, visualization and integrated discovery (DAVID, Dennis *et al.*, 2003) for these 22 genes are shown in Table S5.1 for genes negatively associated with survival, and in Table S5.2 for genes positively associated with survival.

Table S3: Genes significantly modulated by methylation as identified by the iBAG$_{\text{unified}}$ model. Genes in boldface are negatively modulated by their corresponding methylation features; genes in regular font are positively modulated by their corresponding methylation features

Genes	Genes	Genes	Genes	Genes	Genes
ARFRP1	**TSPAN15**	**IL11RA**	**TXNL4B**	**BNIP2**	**CREG1**
**TCIRG1**	VAX2	ITGAM	**SP3**	**CD84**	DLC1
TRPC4AP	LGALS3BP	PBRM1	**SP100**	PLOD3	**PGCP**
**GAS6**	**LLGL2**	**STEAP3**	BPHL	**MAP3K14**	**TAB1**
**FAM50B**	**LYN**	**ECHDC2**	**STAT6**	**CCRL2**	**SPON2**
**GNL3**	**TM4SF1**	**KLHL26**	**BST2**	PAPSS1	**CAP2**
**GFPT1**	**MUT**	**PPP4C**	**BTD**	**DIRAS3**	**PLA2G16**
STEAP1	**MYO1C**	GIMAP5	**NR2F2**	**CCT6A**	**CHI3L1**
**DKK3**	**NAGA**	**IMPACT**	**ACTG2**	**OSMR**	C16orf42
**ZNF544**	**NCF2**	**OTUB1**	**UFD1L**	**LRRFIP1**	CNGA3
**GRB10**	**NDN**	FAR2	**FSD1**	**TRIP4**	**PACRG**
**ANK3**	**ATIC**	PRODH	**RDBP**	**PPT2**	**CSTA**
**BZW2**	**MRPS2**	**TMEM159**	FAM57A	**CDYL**	**GRIK5**
**PCP4**	**PYCR1**	**RPP21**	ENTPD1	C16orf80	**C1RL**
**RAP1A**	**DENND2D**	**EIF2B1**	SNX10	**RASL12**	**RARRES2**
**FAM128B**	**KIAA0040**	**AHR**	**GPSM2**	**ZDHHC3**	RELA
**C2orf44**	**ESPL1**	EML1	**PILRA**	PDE8A	RGS3
**CAPN2**	DHX38	**ENDOG**	**HIST1H1D**	**TRAPPC2L**	**APOBEC3G**
**CDC45**	PSMD6	**FCER1G**	**MNX1**	CAB39	**RPS2**
**SH3BGRL3**	**NUAK1**	**FHIT**	**HOXA1**	**PFDN2**	CFB
**CASP1**	PDLIM2	**FOXD1**	**HSPA1A**	PGM3	**SNAP91**
MT4	ZGPAT	**CFI**	**PLA2G5**	SFRP4	**MVP**
SARM1	**IGFBP6**	**PLEK**	**SGCB**	**CAV2**	
**SEL1L3**	**IL1B**	**TREM2**	**ST3GAL4**	**CBFB**	

Table S4.1: Number of significant genes obtained by the iBAG$_{\text{unified}}$ model with false discovery rate (FDR) = 0.05

Characteristic	Genes with type M effects		Genes with type $\bar{M}$ effects	
	Significant	Nonsignificant	Significant	Nonsignificant
Positively associated with survival	8	146	58	446
Negatively associated with survival	14	180	49	447

Table S4.2: Number of significant genes by obtained by the ADD model with false discovery rate (FDR) = 0.05

Characteristic	Genes obtained by methylation		Genes obtained by gene expression	
	Significant	Nonsignificant	Significant	Nonsignificant
Positively associated with survival	15	171	39	423
Negatively associated with survival	7	155	39	499

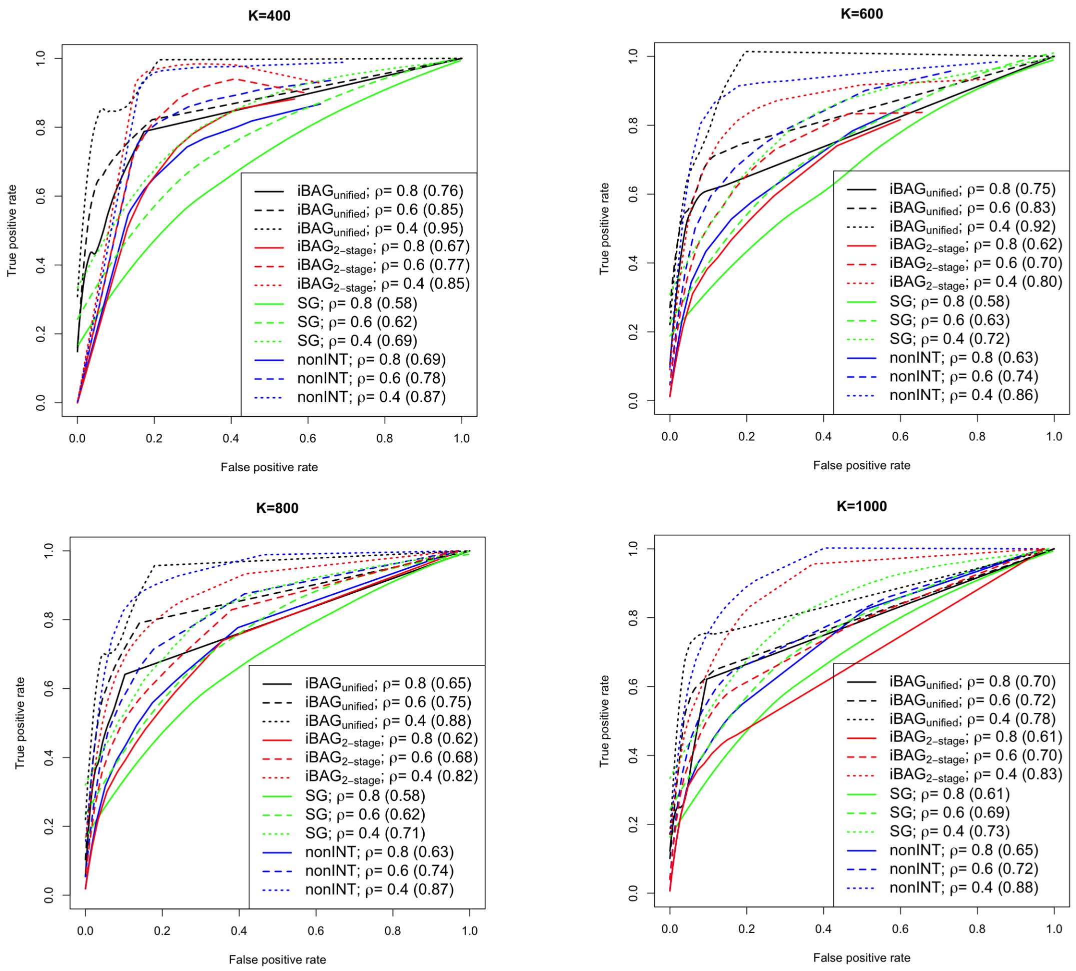

Figure S2.1: ROC curves of the true positive rate versus the false positive rate for discovering genes with only type M effects (effects modulated only by methylation), using the nonINT, SG, iBAG$_{\text{2-stage}}$, and iBAG$_{\text{unified}}$ models. The values in parentheses are the AUCs corresponding to the ROC curves.

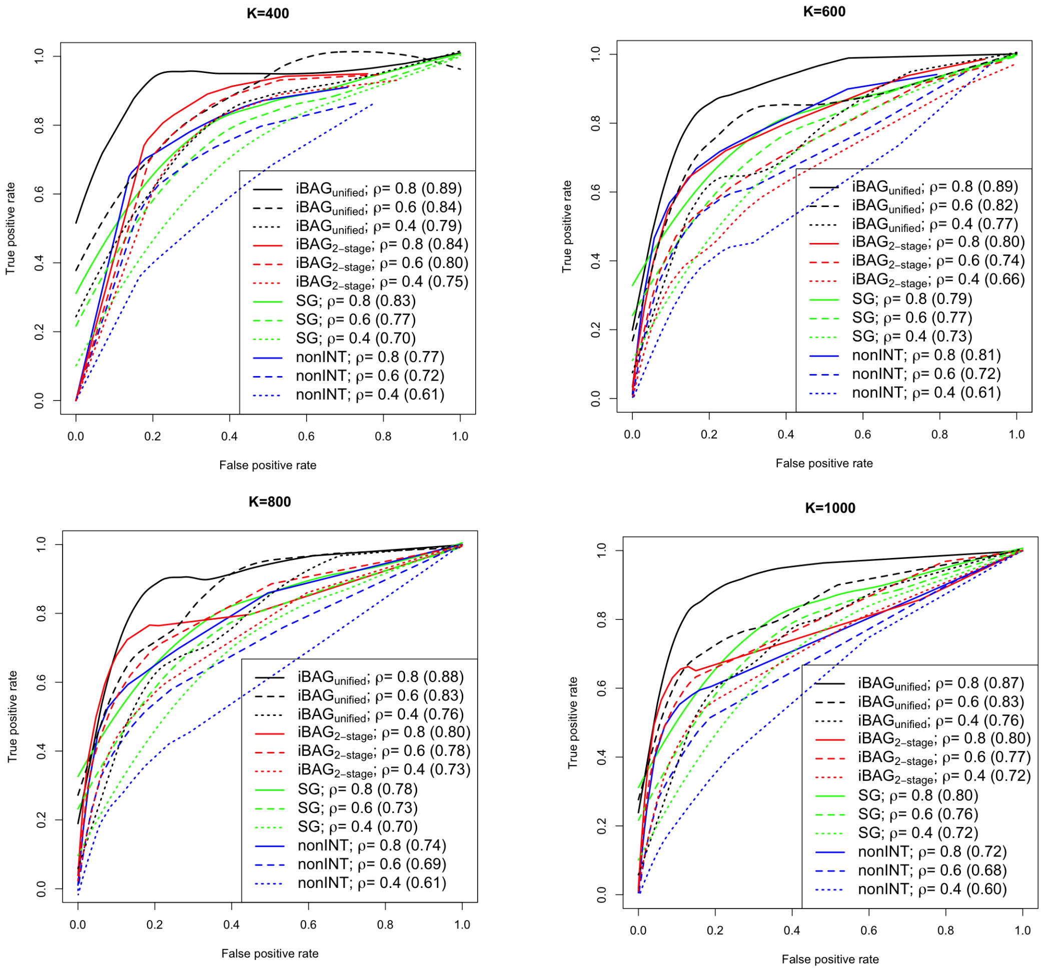

Figure S2.2: ROC curves of the true positive rate versus the false positive rate for discovering genes with only type $\bar{M}$ effects (effects modulated only by other mechanisms), using the nonINT, SG, iBAG$_{\text{2-stage}}$, and iBAG$_{\text{unified}}$ models. The values in parentheses are the AUCs corresponding to the ROC curves.

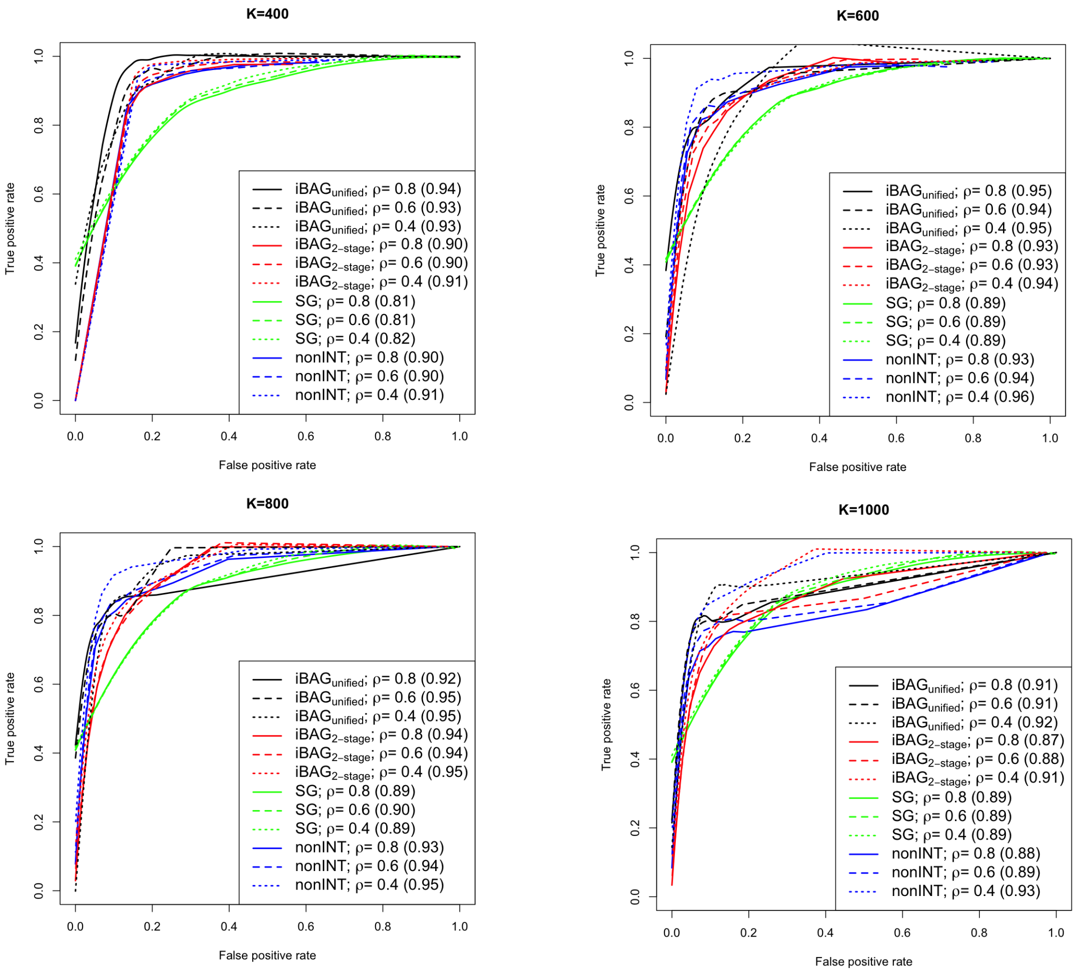

Figure S2.3: ROC curves of the true positive rate versus the false positive rate for discovering the genes with type $M + \bar{M}$ effects (effects modulated by both methylation and other mechanisms), using the nonINT, SG, $\text{iBAG}_{\text{2-stage}}$ and $\text{iBAG}_{\text{unified}}$ models. The values in parentheses are the AUCs corresponding to the ROC curves.

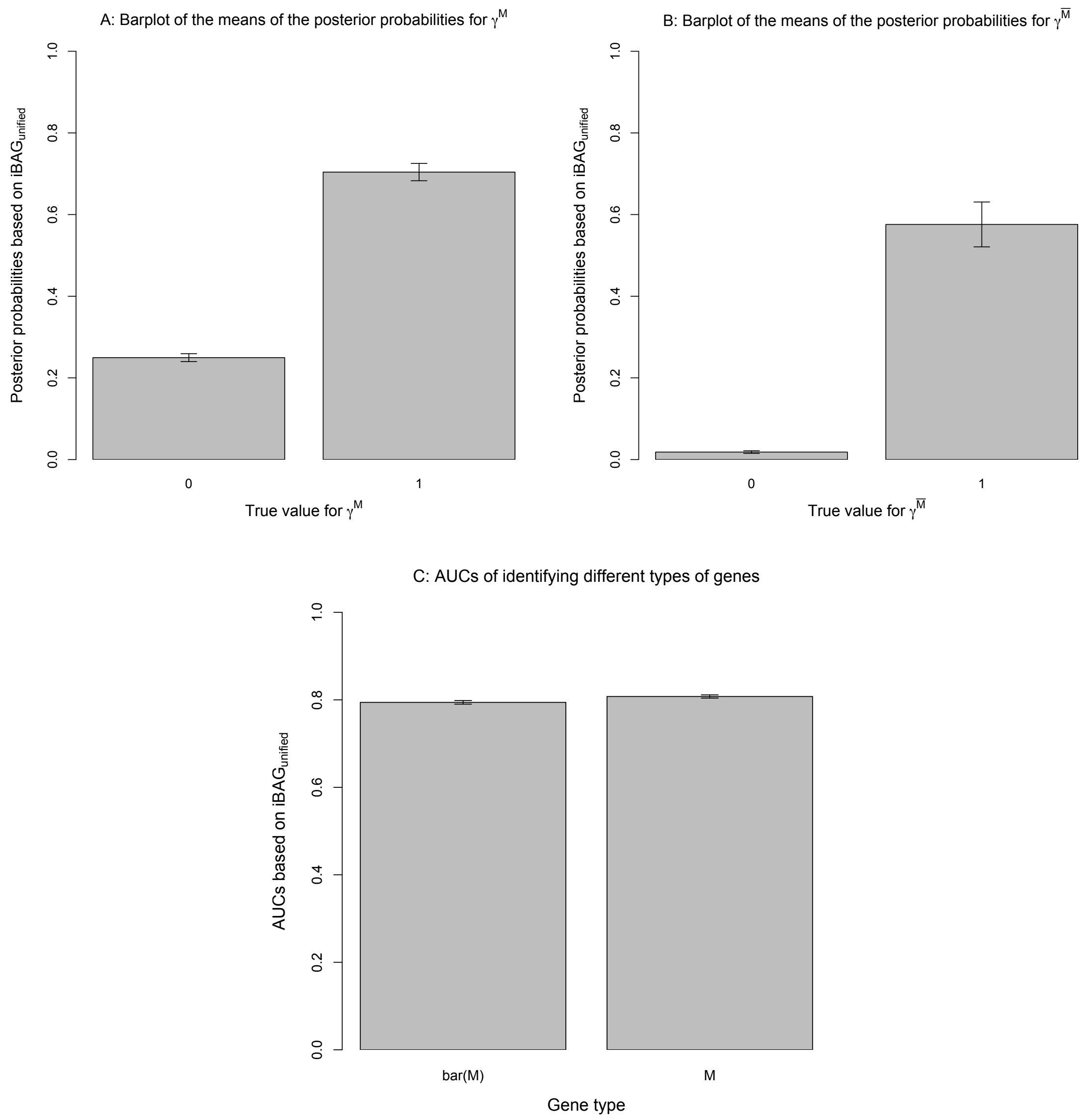

Figure S3: Barplot with standard errors for the means of the posterior probabilities of gene expression effects (panel A for γ^M and panel B for $\gamma^{\bar{M}}$) estimated by the iBAG$_{\text{unified}}$ model and the area under the ROC curves for identifying type M genes and type $\bar{M}$ genes (panel C) from 100 datasets generated for $K = 1000$ and $\rho = -0.6$. Type $\bar{M}$ genes: genes with nonzero gene expression effects modulated by other genomic changes; Type M genes: genes with nonzero gene expression effects modulated by methylation.

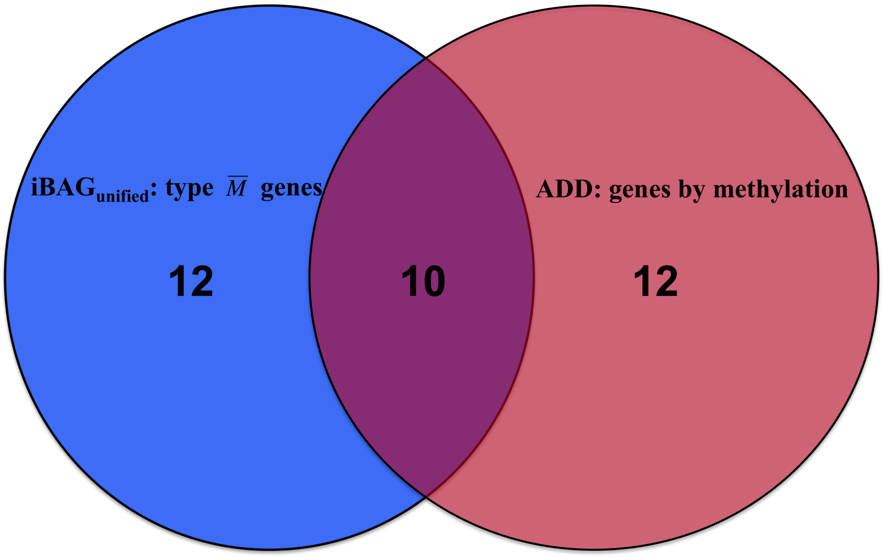

Figure S4.1: Venn diagram comparing type M genes identified by the iBAG$_{\text{unified}}$ model, and genes identified by methylation using the ADD model (modeling both gene expression and methylation information and assuming their effects on patients' survival times are additive)

Twelve genes obtained by both iBAG$_{\text{unified}}$ and ADD models: CAP2, POLR3C, CNGA3, FKBP1B, RNF115, HOXA1, PCP4, CYB5R2, TK1, C2orf44, SF3B5, CASP4

Ten genes obtained only by the iBAG$_{\text{unified}}$ model: SPON2, DPP4, GPR116, SARM1, RBBP4, SMURF2, C1QA, UFD1L, CBFB, MVP

Ten genes obtained only by the ADD model: E2F4, DBB1, ANK3, IL11RA, NDN, WDR45L, PPAN, SGCB, NIPSNAP1, OSMR

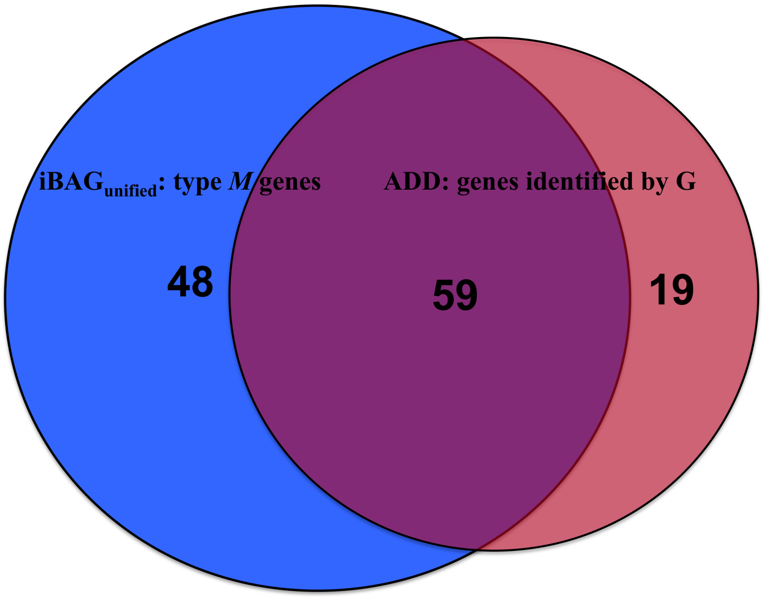

Figure S4.2: Venn diagram comparing type $\bar{M}$ genes identified by the iBAG$_{\text{unified}}$ model, and genes identified by methylation using the ADD model (modeling both gene expression and methylation information and assuming their effects on patients' survival times are additive)

Fifty-nine genes obtained by both iBAG$_{\text{unified}}$ and ADD models: LPCAT3, TRIB1, TAB1, DCTN2, FARS2, RPP40, OS9, SLC27A5, TMEM115, POLI , NXPH3, ADCY8, SEP10, FCER1G, FHIT, KIAA1012, FRAT2, NPTXR, PISD, CCDC19, ZNF544, GNS, GRIK5, GSTM3, MNX1, HOXC4, IL10RB, INPPL1, KARS, MAP3K10, ATIC, MRTO4, VPS28, PDE8A, ENPP2, PHKG1, NDE1, FBXO34, ARHGEF10L, ADI1, BIN3, BEX4, EPB41L5, RGS3, RPS4X, SP100, SREBF1, SURF2, TOP1, UBE2V2, ADIPOR2, SLC24A6, ZNF430, NPRL3, ZNF528, CSDA, RPL23, KIAA0141, HS3ST2

Forty-eight genes obtained only by the iBAG$_{\text{unified}}$ model: PEMT, PNPLA6, C16orf42, CLTC, CNGA3, CSTA, E2F4, AIM1, STX2, ZBTB1, FAM50B, DKK3, HSPA1A, IGBP1, IPW, ITPR2, LRP3, NCF2, ACO1, PABPC3, WBP11, PFDN2, POLR2H, RC3H2, C12orf35, PPP2R2A, GIMAP5, AMBRA1, UBFD1, ELOVL5, RPE, CFB, PLEK, PORCN, SP3, STAT6, TACC1, TLE1, VDAC3, SLC39A7, SH3BGRL3, MT4, RUVBL1, HERC2, DIRAS3, EIF1AY, VAPB, SNCAIP

Nineteen genes obtained only by the ADD model: SF3B4, PIAS3, EIF2B1, EIF5A, FLRT2, ODZ4, ADAMDEC1, GNL1, HSF2, HAUS2, H2AFY2, WSB2, WDR45L, NIT2, PTPRG, STAU1, DDA1, GPR172A, HDAC9

Table S5.1: Functional analysis results using the database for annotation, visualization and integrated discovery genes (DAVID, Dennis *et al.*, 2003) for the 18 genes with significant NEGATIVE methylation-modulated effects, as identified by the $\text{iBAG}_{\text{unified}}$ model.
Reading left to right: the first column shows the different categories of gene ontology clusters; the second column shows the gene ontology terms with which these genes are associated; the third and fourth columns show the number and the percentage of genes within this gene ontology cluster; the fifth column shows the p-value for the enrichment of the gene ontology cluster; the last column shows the Entrez GeneID for each of the genes belonging to this cluster.

Category	Term	Count	% Genes	P-value	Genes (Entrez GeneID)
Biological Process					
GOTERM_BP_ALL	GO:0000902 cell morphogenesis	3	21.42	0.035	HOXA1, CAP2, SPON2
GOTERM_BP_ALL	GO:0032989 cellular component morphogenesis	3	21.42	0.043	HOXA1, CAP2, SPON2
GOTERM_BP_ALL	GO:0009653 anatomical structure morphogenesis	4	28.57	0.075	HOXA1, CAP2, FKBP1A, SPON2
GOTERM_BP_ALL	GO:0007178 transmembrane receptor protein serine/threonine kinase signaling pathway	2	14.28	0.084	FKBP1A, SMURF2
GOTERM_BP_ALL	GO:0007411 axon guidance	2	14.28	0.087	HOXA1, SPON2
Molecular Function					
GOTERM_MF_ALL	GO:0005217 intracellular ligand-gated ion channel activity	2	14.28	0.013	FKBP1A, CNGA3
Functional Annotation Clustering					
Annotation Cluster 1	Enrichment Score: 0.373				
SP_PIR_KEYWORDS	nucleus	5	35.71	0.324	HOXA1, RBBP4, SMURF2, POLR3C, MVP
GOTERM_BP_FAT	GO:0045449 regulation of transcription	4	28.57	0.414	HOXA1, RBBP4, SMURF2, POLR3C
SP_PIR_KEYWORDS	Transcription	3	21.43	0.415	HOXA1, RBBP4, POLR3C
GOTERM_BP_FAT	GO:0006350 transcription	3	21.43	0.577	HOXA1, RBBP4, POLR3C
Annotation Cluster 2	Enrichment Score: 0.165				
SP_PIR_KEYWORDS	cell membrane	3	21.42	0.446	CAP2, SMURF2, GPR116
GOTERM_CC_FAT	GO:0005886 plasma membrane	3	21.42	0.843	CAP2, SMURF2, GPR116
SP_PIR_KEYWORDS	membrane	4	28.57	0.847	CAP2, SMURF2, CNGA3, GPR116

Table S5.2: Functional analysis results using the database for annotation, visualization and integrated discovery genes (DAVID) for the 8 genes with significant POSITIVE methylation-modulated effects, as identified by the $\text{iBAG}_{\text{unified}}$ model. (Same column groupings as in Table S5.1)

Category	Term	Count	% Genes	P-value	Genes (Gene Symbol)
Biological Process					
GOTERM_BP_ALL	GO:0002376 immune system process	4	50	0.0059	C1QA, SARM1, DPP4, CBFB
GOTERM_BP_ALL	GO:0045087 innate immune response	2	25	0.057	C1QA, SARM1
GOTERM_BP_ALL	GO:0006508 proteolysis	3	37.5	0.068	C1QA, UFD1L, DPP4
GOTERM_BP_ALL	GO:0046649 lymphocyte activation	2	25	0.081	DPP4, CBFB
GOTERM_BP_ALL	GO:0050776 regulation of immune response	2	25	0.092	C1QA, DPP4
GOTERM_BP_ALL	GO:0042221 response to chemical stimulus	3	37.5	0.096	SARM1, UFD1L, DPP4
GOTERM_BP_ALL	GO:0045321 leukocyte activation	2	25	0.098	DPP4, CBFB
Cellular Component					
GOTERM_CC_ALL	GO:0043231 intracellular membrane-bounded organelle	7	87.5	0.015	C1QA, SARM1, UFD1L, PCP4, SF3B5, DPP4, CBFB
GOTERM_CC_ALL	GO:0043227 membrane-bounded organelle	7	87.5	0.016	C1QA, SARM1, UFD1L, PCP4, SF3B5, DPP4, CBFB
GOTERM_CC_ALL	GO:0043229 intracellular organelle	7	87.5	0.032	C1QA, SARM1, UFD1L, PCP4, SF3B5, DPP4, CBFB
GOTERM_CC_ALL	GO:0043226 organelle	7	87.5	0.032	C1QA, SARM1, UFD1L, PCP4, SF3B5, DPP4, CBFB
GOTERM_CC_ALL	GO:0044444 cytoplasmic part	5	62.5	0.076	C1QA, SARM1, UFD1L, PCP4, DPP4
GOTERM_CC_ALL	GO:0044424 intracellular part	7	87.5	0.088	C1QA, SARM1, UFD1L, PCP4, SF3B5, DPP4, CBFB
Molecular Function					
GOTERM_MF_ALL	GO:0005515 protein binding	5	62.5	0.084	SARM1, RNF115, UFD1L, DPP4, CBFB
Functional Annotation Clustering					
Annotation Cluster 1	Enrichment Score: 0.546				
SP_PIR_KEYWORDS	acetylation	4	50	0.058	RNF115, UFD1L, SF3B5, CBFB
SP_PIR_KEYWORDS	nucleus	3	37.5	0.484	UFD1L, SF3B5, CBFB
SP_PIR_KEYWORDS	phosphoprotein	3	37.5	0.810	UFD1L, SF3B5, CBFB
Annotation Cluster 2	Enrichment Score: 0.108				
SP_PIR_KEYWORDS	polymorphism	5	62.5	0.711	C1QA, SARM1, RNF115, UFD1L, CBFB
UP_SEQ_FEATURE	sequence variant	5	62.5	0.761	C1QA, SARM1, RNF115, UFD1L, CBFB
SP_PIR_KEYWORDS	alternative splicing	3	37.5	0.827	SARM1, UFD1L, CBFB
UP_SEQ_FEATURE	splice variant	3	37.5	0.828	SARM1, UFD1L, CBFB

MCMC diagnostics: To check the convergence of the $\text{iBAG}_{\text{unified}}$ model, we run two MCMC chains with different starting values, and obtain the trace plots and Gelman and Rubin's diagnostic statistics by iterations (Gelman and Rubin, 1992), which are denoted as Gelman and Rubin plots, for important parameters in the $\text{iBAG}_{\text{unified}}$ model (Figure S5). Gelman and Rubin's diagnostic statistics measure the convergence of MCMC samples for multiple chains by comparing the variance within chains to the variance between chains. Values substantially above 1 indicate a lack of convergence. The plots on the left side of Figure S5 are the trace plots of important parameters (those controlling the overall sparsity of the $\text{iBAG}_{\text{unified}}$ models and those with meaningful biological interpretations, e.g., shrinkage parameters for gene expression effects (λ^M and $\lambda^{\bar{M}}$), some of the significant gene expression effects, and some of the significant methylation effects) in the applications of the $\text{iBAG}_{\text{unified}}$ model. The three plots on the right side of Figure S5 are Gelman and Rubin plots that correspond to the trace plots on the left. In each Gelman and Rubin plot, the x-axis depicts the iteration number and the y-axis depicts the values of Gelman and Rubin's diagnostic statistics. The solid line in the figure represents the median estimates, and the dashed line represents the 97.5% quantile of Gelman and Rubin's diagnostic statistics along iterations. From Figure S5, we observe that due to the closed form of the conditional posterior distributions for the $\text{iBAG}_{\text{unified}}$ model – the parameters usually converge very fast and admit good mixing properties. (Gelman and Rubin's diagnostic statistic usually converges to 1 after about 2000 iterations). In addition, a directory with the traceplots of all our parameters along with a brief description is available at the following link: http://odin.mdacc.tmc.edu/~vbaladan/Veera_Home _Page/Software _files/TracePlots.zip

References

Andrews, DR and Mallows, CL (1974). Scale mixtures of normal distributions. *J. R. Statist. Soc. B*, **36**, 99-102.

Dennis G Jr, Sherman BT, Hosack DA, *et al.* (2003), DAVID: Database for Annotation, Visualization, and Integrated Discovery. *Genome Biol.*, **4(5)**:P3.

Gelman, A and Rubin, DB (1992) Inference from iterative simulation using multiple sequences, *Stat. Sci.*, **7**, 457-511.

Griffin, JE and Brown, PJ (2010) Inference with normal-gamma prior distributions in regression problems. *Bayesian Analysis*, **5 (1)**. pp. 171-188.

Griffin, JE and Brown, PJ (2011). Bayesian hyper-lassos with non-convex penalization. *Aust. N. Z. J. Stat.*, **53**, 423442.

Knight, K and Fu, W (2000). Asymptotics for lasso-type estimators. *Ann. Stat.*, **28(5)**:1356 1378

Park, T and Casella, G (2008). The Bayesian Lasso. *J. Am. Stat. Assoc.*, **103**, 681-686.

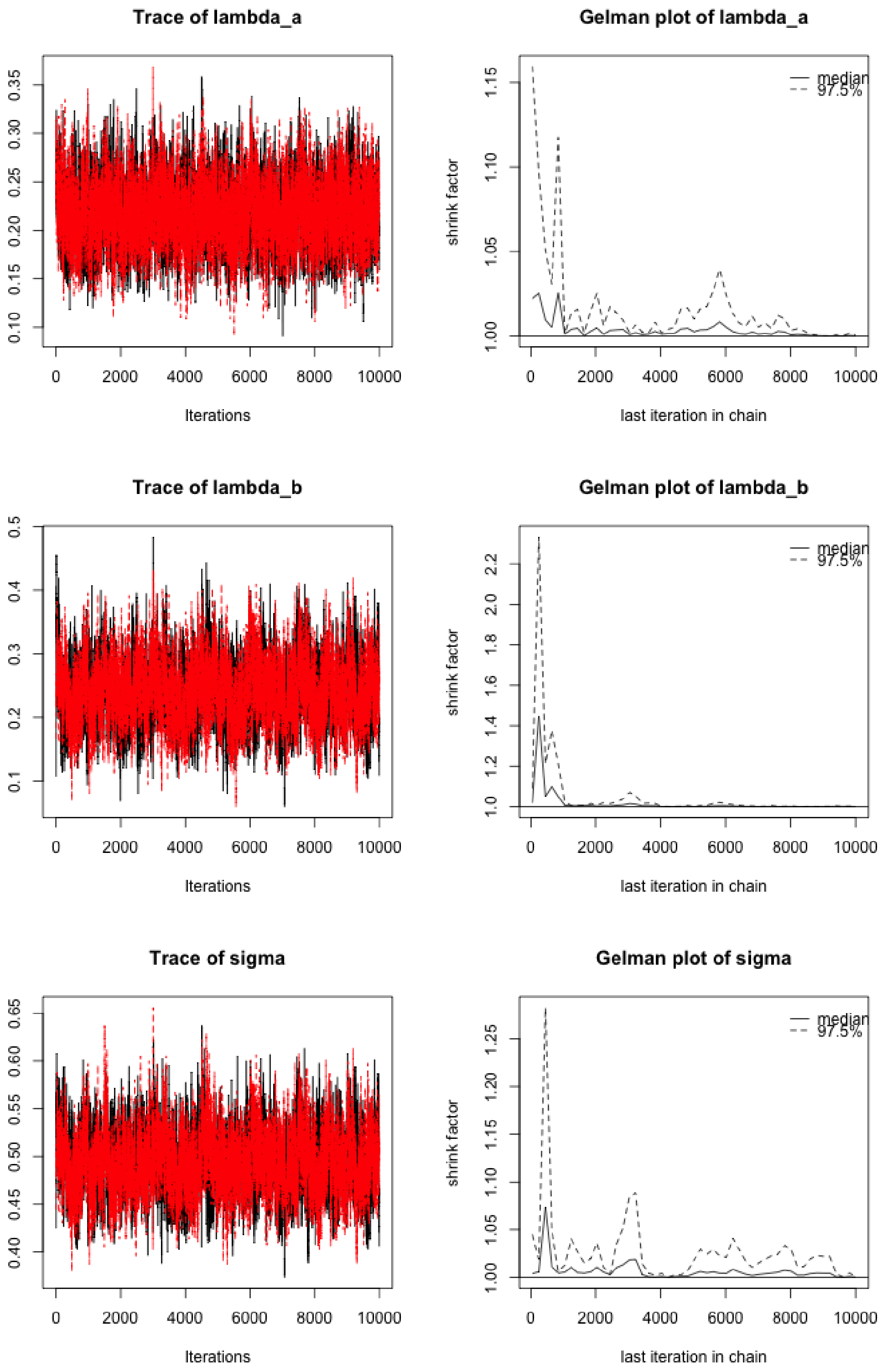

Figure S5-I : Trace plots and Gelman and Rubin plots of λ_a, λ_b, and σ after applying the iBAG model to the GBM dataset

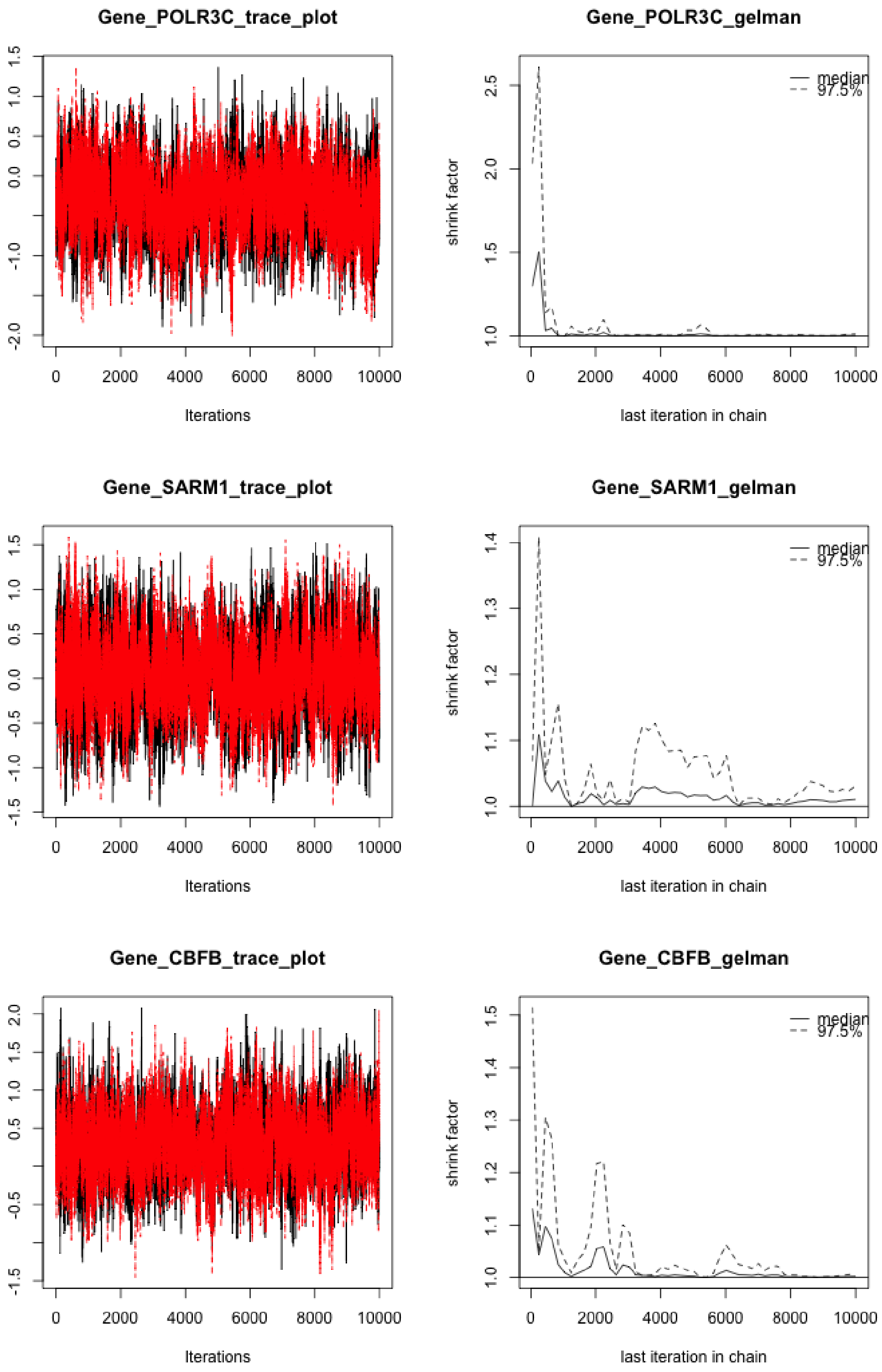

Figure S5-II: Trace plots and Gelman and Rubin plots of γ^M estimation for significant genes POLR3C, SARM1, and CBFB respectively, from top to bottom

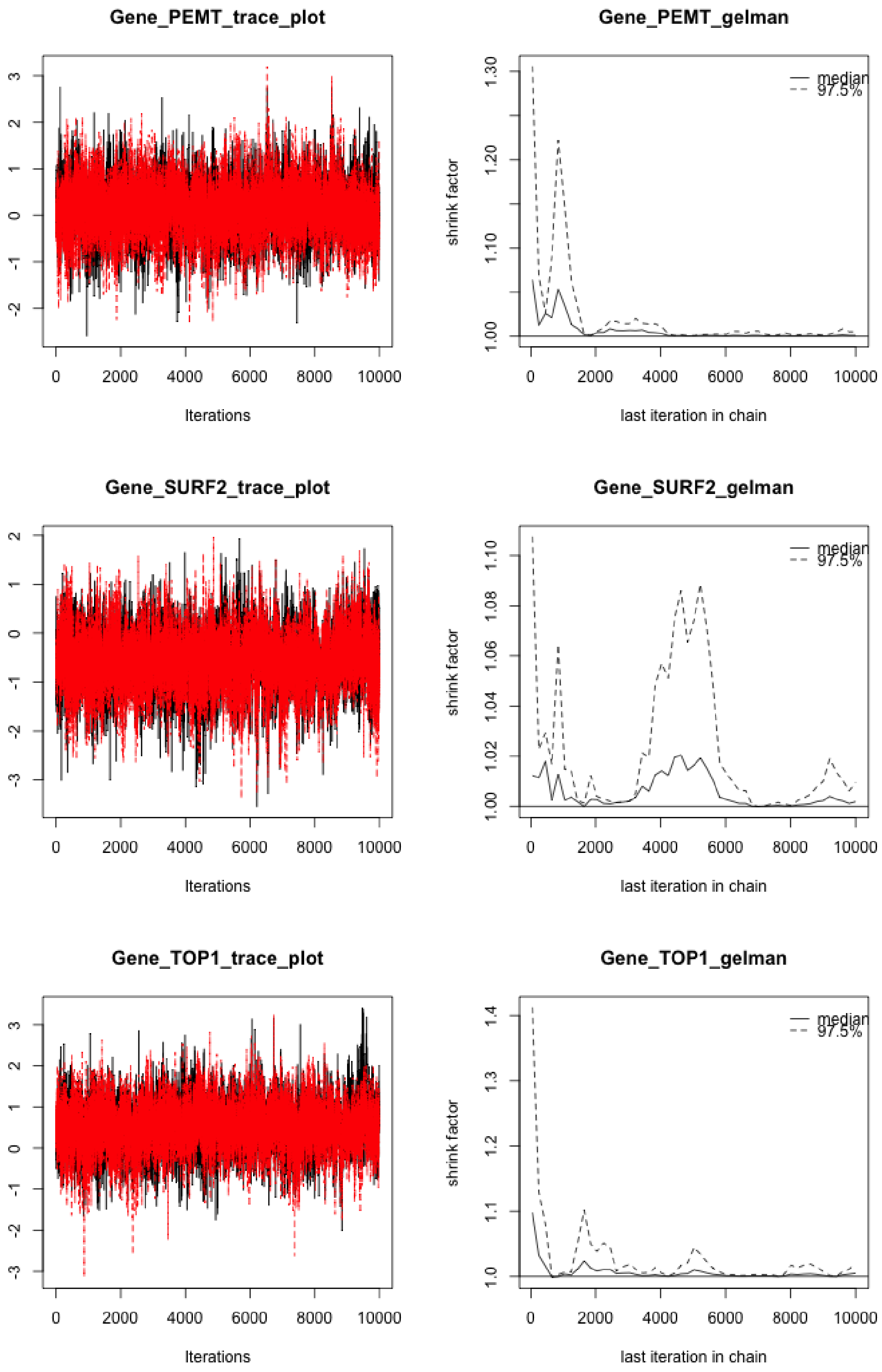

Figure S5-III: Trace plots and Gelman and Rubin plots of $\gamma^{\bar{M}}$ estimation for significant genes PEMT, SURF2, and TOP1 respectively, from top to bottom

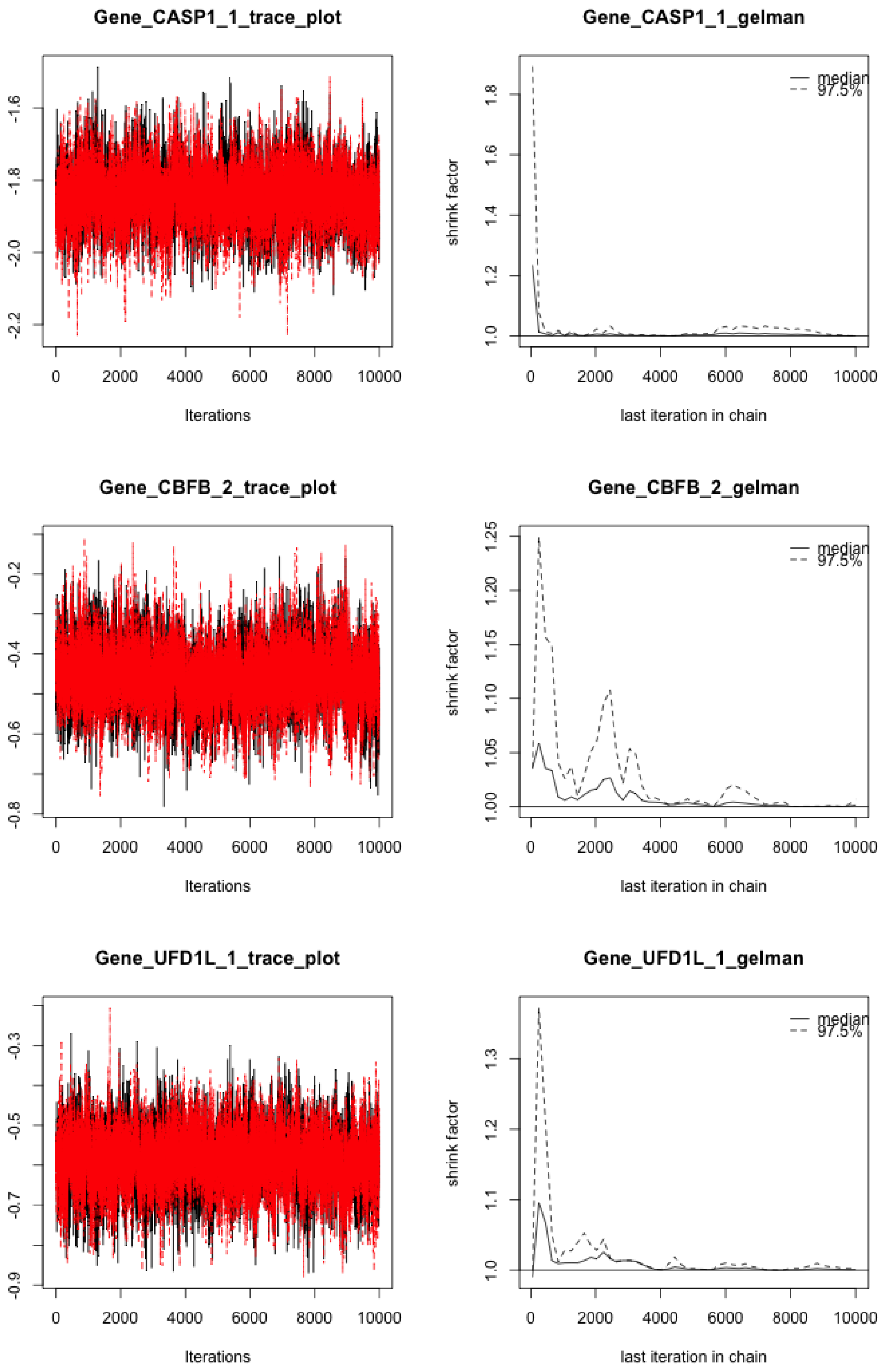

Figure S5-IV: Trace plots and Gelman and Rubin plots of significant ω estimation for genes CASP1, CBFB, and UFD1L respectively, from top to bottom
